# LPS Primes Brain Responsiveness to High Mobility Group Box-1 Protein

**DOI:** 10.3390/ph14060558

**Published:** 2021-06-11

**Authors:** Verena Peek, Lois M. Harden, Jelena Damm, Ferial Aslani, Stephan Leisengang, Joachim Roth, Rüdiger Gerstberger, Marita Meurer, Maren von Köckritz-Blickwede, Sabine Schulz, Bernhard Spengler, Christoph Rummel

**Affiliations:** 1 Institute of Veterinary Physiology and Biochemistry, Justus Liebig University Giessen, 35392 Giessen, Germany; verenapeek@web.de (V.P.); jelena-damm@gmx.de (J.D.); Stephan.Leisengang@vetmed.uni-giessen.de (S.L.); Joachim.Roth@vetmed.uni-giessen.de (J.R.); Ruediger.Gerstberger@vetmed.uni-giessen.de (R.G.); 2 Brain Function Research Group, School of Physiology, Faculty of Health Sciences, University of Witwatersrand, Johannesburg 2193, South Africa; Lois.Harden@wits.ac.za; 3 Institute of Anatomy and Cell Biology of the Medical Faculty, Justus Liebig University, 35392 Giessen, Germany; ferial.aslani@gmail.com; 4 Department of Biochemistry, University of Veterinary Medicine Hannover, 30559 Hannover, Germany and Research Center for Emerging Infections and Zoonoses (RIZ), University of Veterinary Medicine Hannover, 30559 Hannover, Germany; Marita.Meurer@tiho-hannover.de (M.M.); maren.von.koeckritz-blickwede@tiho-hannover.de (M.v.K.-B.); 5 Institute of Inorganic and Analytical Chemistry, Justus Liebig University Giessen, 35392 Giessen, Germany; Sabine.Schulz@anorg.Chemie.uni-giessen.de (S.S.); bernhard.spengler@anorg.chemie.uni-giessen.de (B.S.)

**Keywords:** cytokines, lipopolysaccharide, high mobility group box-1 protein, circumventricular organs, septic-like inflammation

## Abstract

High mobility group box (HMGB)1 action contributes to late phases of sepsis, but the effects of increased endogenous plasma HMGB1 levels on brain cells during inflammation are unclear. Here, we aimed to further investigate the role of HMGB1 in the brain during septic-like lipopolysaccharide-induced inflammation in rats (LPS, 10 mg/kg, i.p.). HMGB-1 mRNA expression and release were measured in the periphery/brain by RT-PCR, immunohistochemistry and ELISA. In vitro experiments with disulfide-HMGB1 in primary neuro-glial cell cultures of the area postrema (AP), a circumventricular organ with a leaky blood–brain barrier and direct access to circulating mediators like HMGB1 and LPS, were performed to determine the direct influence of HMGB1 on this pivotal brain structure for immune-to-brain communication. Indeed, HMGB1 plasma levels stayed elevated after LPS injection. Immunohistochemistry of brains and AP cultures confirmed LPS-stimulated cytoplasmatic translocation of HMGB1 indicative of local HMGB1 release. Moreover, disulfide-HMGB1 stimulation induced nuclear factor (NF)-κB activation and a significant release of interleukin-6, but not tumor necrosis factor α, into AP culture supernatants. However, only a few AP cells directly responded to HMGB1 with increased intracellular calcium concentration. Interestingly, priming with LPS induced a seven-fold higher percentage of responsive cells to HMGB1. We conclude that, as a humoral and local mediator, HMGB1 enhances brain inflammatory responses, after LPS priming, linked to sustained sepsis symptoms.

## 1. Introduction

High mobility group box (HMGB)1 is a ubiquitous nuclear DNA-binding protein, which is involved in modulating chromatin structure [1], DNA replication, repair, and transcription [2,3]. When actively or passively released into the extracellular space by necrotic or stimulated cells like macrophages, HMGB1 becomes an endogenous danger associated molecular pattern (DAMP) engaged in a variety of functions including immunity, cell proliferation or cell death [3,4]. Its release by immune cells, neurons, astrocytes and microglia or other cell types in the periphery is accompanied by translocation from the nucleus to the cytoplasm [5,6,7]. Moreover, these cells not only release HMGB1, they can also be stimulated by it to release immune mediators including the pro-inflammatory cytokines interleukin (IL-)6 and/or tumor necrosis factor (TNF)α [6,8,9,10]. Importantly, HMGB1′s extracellular mode of action highly depends on its redox status. The fully reduced form exhibits chemotactic function, the disulfide form has cytokine activity, and the fully oxidized sulfonyl HMGB1 seems to be part of a negative feedback mechanism involved in the resolution of inflammation [11,12]. HMGB1 signals via toll-like receptor (TLR)4 or the receptor for advanced glycation end products (RAGE) and is known to induce nuclear factor (NF)κB activation [4].

Systemic inflammation, induced by lipopolysaccharide (LPS), a compound of Gram-negative bacterial cell walls, can exacerbate ongoing brain pathologies including neuro-degeneration [13] or stroke [14,15]. Experimentally, the bacterial mimetic LPS is one of the most commonly used models to study severe systemic inflammation, as it reflects several components observed during sepsis like the systemic inflammatory response syndrome (also termed septic-like inflammation) in humans and animals [16]. It is now well established that DAMPs, such as HMGB1, play a crucial role in inflammatory responses, and in cases where predisposing factors and systemic inflammation occur with concurrent brain pathologies [17], it may worsen the progression of the disease [18,19]. Indeed, in an animal model of post-stroke infection, HMGB1 was shown to aggravate inflammation, enhance deterioration of behavioral outcomes, and infarct expansion [20]. In line with these findings of worsening disease outcomes in post-stroke infection, inhibiting the action of HMGB1 has been shown to have high therapeutic potential for neurological diseases, e.g., neurodegeneration or stroke [18,19,21]. Moreover, a role for HMGB1 as a mediator of the latter phase of septic inflammation, induced by a high dose of LPS, has been demonstrated in various studies [22]. Using HMGB1-neutralizing strategies in rodents, these studies showed reduced inflammation [22,23,24,25,26]. Long-lasting high circulating HMGB1 concentrations have also been reported in human septic patients compared to heathy controls [22,27], and patients that died of septic shock had higher HMGB1 levels [27]. Moreover, high plasma HMGB1 levels occur in a variety of other disease states and situations including traumatic brain injury, as recently confirmed in a systematic review of human and animal studies [28]. Recently, HMGB1 has even been proposed as a common biomarker for early prediction and progression of traumatic brain injury (TBI), neuroinflammation, and cognitive dysfunctions [29]. For example, human plasma HMGB1 levels were associated with mild cognitive deficits and reduced cortical thickness [30]. Moreover, during septic inflammation induced by cecal ligation and puncture, HMGB1 was demonstrated to convey long-term memory impairments in surviving animals [26]. The authors used neutralizing HMGB1 antibodies to inhibit memory impairments and i.p. daily injection of 500 µg HMGB1 for three weeks to induce cognitive impairments.

A role for HMGB1, at least the disulfide form [6], in mediating LPS-induced sickness responses has been suggested following the observation that intracerebroventricular (icv) injections of HMGB1 induced typical symptoms of sickness including anorexia, taste aversion and fever and systemic neutralization of HMGB1 inhibited LPS-induced anorexia, suggesting its role in LPS-induced sickness responses [31,32]. Interestingly, subsequent studies have shown that exogenous icv HMGB1-pretreatment or endogenously produced HMGB1 after induction of sterile inflammation (turpentine) exacerbated LPS-induced reduced social exploration [6] or fever [33], respectively. Similarly, even severe psychological stress or aging can induce the release of HMGB1 from microglia and neurons in the brain leading to a brain inflammatory response accompanied by depressive-like symptoms [34,35,36] and to a primed, enhanced immune and sickness response in aged rats to subsequent *Escherichia coli* infection [37].

Despite the evidence in support of HMGB1 acting as a central mediator of brain inflammatory responses, the immune-to-brain communication pathways by which in-creased endogenous plasma HMGB1 levels act on the brain remains only partially under-stood. In addition to the potential role of endothelial cells in transferring inflammatory information to the brain, as previously shown for other large proteins like IL-6 [38,39], circumventricular organs (CVOs), brain structures with a leaky blood–brain barrier, can also directly detect circulating inflammatory mediators [40] and induce de novo synthesis of secondary lipophilic mediators, such as prostaglandins, to convey information into deeper brain structures [41]. Moreover, the sensory (s)CVOs, like the area postrema (AP), contain neurons that also transfer inflammatory information, via neuronal signaling, to further brain sites. Indeed, brain cells within the sCVOs show direct activation by LPS or cytokines, such as TNFα, as previously shown using calcium imaging in primary neuro-glial cell cultures of the AP [42,43].

Here, for the first time, we aim to investigate the potential role of HMGB1 in immune-to-brain communication within the AP. To this end, we first determined whether HMGB1 was released in vivo during LPS-induced septic-like inflammation and tested its potential to induce IL-6 and TNFα production, related NFκB signaling, release of fatty acids and direct cell activation in AP cells using bioassays, immunohistochemistry, LC MS/MS and calcium imaging, respectively. While previous studies have revealed that HMGB1 may prime brain cells for subsequent inflammatory insults [6,35,37], we now add to this concept and propose that LPS-induced inflammation sensitizes brain cells in the AP to become directly responsive to subsequent endogenous HMGB1 release in the periphery and the brain. Thus, this scenario follows the naturally occurring sequence of septic inflammation with high presence of LPS in the circulation followed by increased brain and plasma HMGB1 levels. To this end, increased levels of circulating HMGB1 can be detected during a variety of conditions, like sepsis, contributing to the vast number of humoral mediators in immune-to-brain communication during systemic inflammatory conditions.

## 2. Results

### 2.1. The Course of the Febrile Response Is Accompanied by Prolonged Circulating HMGB1 Levels but Not Plasma IL-6 and TNF Levels during Septic-Like LPS-Induced Systemic Inflammation

**Figure 1:**
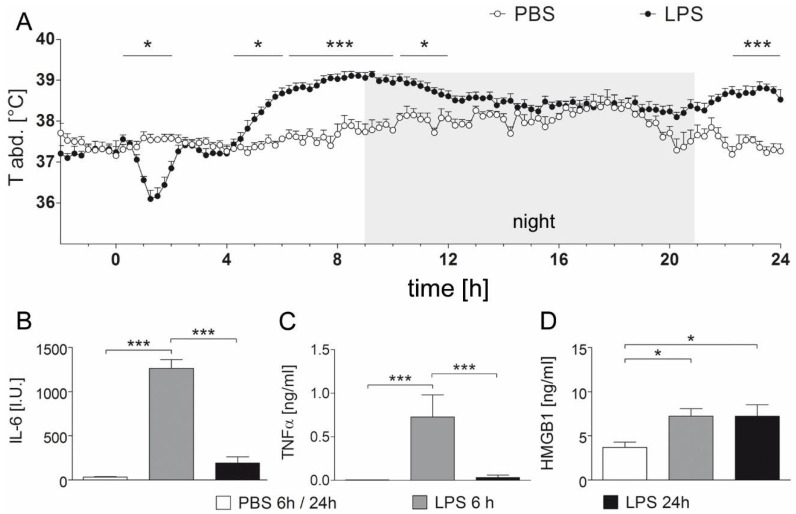
LPS-induced febrile response and increase of circulating mediators. (**A**) Abdominal temperature [°C] was recorded for 24 h after injection of LPS (10 mg/kg, n = 5) or PBS (n = 4) by intra-abdominal radio transmitters. After initial hypothermia (1.25 h i.p.), LPS-injected rats responded with significantly enhanced body core temperature from 4.25 h to 12 h i.p. and from 22.25 h to 24 h compared to PBS-treated control animals. Two-way repeated measures ANOVA with the between-subject factor treatment and the within-subject factor time followed by Bonferroni correction for multiple comparisons; graphs represent temperature measurements every 15 min, depicted as means ± SEM. * = *p* < 0.05, *** = *p* < 0.0001. (**B**–**D**) After 6 h and 24 h, concentrations of the circulating cytokines IL-6 (**B**) and TNFα (**C**) were detected by specific bioassays, while an ELISA was applied for HMGB1 measurements (**D**). Rats injected with LPS showed significantly increased plasma levels of IL-6 and TNFα at 6 h but not at 24 h after injection. HMGB1 concentrations were significantly elevated compared to PBS treated rats at both time points. 6 h/24 h PBS, n = 7; 6 h LPS, n = 4; 24 h LPS, n = 5; one-way ANOVA followed by Tukey multiple comparison tests; bars represent means ± SEM. * = *p* < 0.05, *** = *p* < 0.001.

Rats receiving LPS (10 mg/kg) showed an initial hypothermia, followed by fever lasting for ~eight hours (4.25–6 h (*p* = 0.048); 6.25–10 h (*p* < 0.0001); 10.25–12 h (*p* = 0.024)) and a second febrile phase 22.25 to 24 h (*p* < 0.0001) after injection compared to phosphate buffered saline (PBS) control counterparts (Figure 1A). Hypothermia (*p* = 0.024) peaked 1.25 h after treatment and turned into fever 4.25 h post injection (i.p.). The highest core body temperature (39.14 °C) measured in the rats occurred 9.25 h after LPS injection. PBS-injected control rats showed a physiological circadian rhythm with a higher core body temperature in the active phase, i.e., at night.

Indicative of the systemic inflammatory response, and as established humoral mediators of fever [38,44], the concentration of the circulating pro-inflammatory cytokines interleukin-6 (IL-6) and tumor necrosis factor α (TNFα) was measured. As expected, plasma IL-6 and TNFα significantly increased six hours after LPS injection versus PBS-treated animals (*p* < 0.001), and almost reached basal levels again 24 h after treatment (Figure 1B,C), while fever was still detected. However, significantly increased circulating HMGB1 was maintained at both 6 and 24 h after injection of LPS compared to PBS-injected control rats (*p* < 0.05), suggesting its potential significance for prolonging the inflammatory response and fever.

### 2.2. HMGB1, RAGE and TLR4 mRNA Expression Are Not Altered in the Hypothalamus and Spleen, While Their Expression Is Reduced in the Liver during LPS-Induced Septic-Like Inflammation

**Figure 2:**
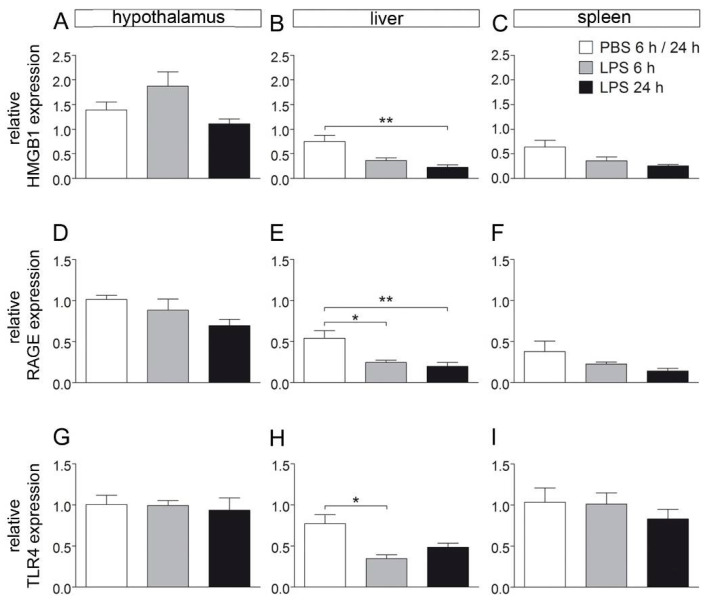
Relative expression of HMGB1 and its receptors in the hypothalamus, liver, and spleen after injection with LPS. Rats were sacrificed 6 or 24 h after LPS injection (10 mg/kg) and brain, liver, and spleen were collected for RT-PCR experiments to investigate expression of HMGB1 (**A**–**C**), RAGE (**D**–**F**), and TLR4 (**G**–**I**). In samples of the hypothalamus and spleen, no effect of LPS treatment was observed on relative expression of investigated genes. However, within the liver, expression of HMGB1 was attenuated 24 h after LPS injection (**B**). Expression of RAGE, a receptor for HMGB1, was reduced 6 and 24 h after injection (**E**), while TLR4 receptor expression was attenuated after 6 h, but not after 24 h. 6 h/24 h PBS, n = 6–7; 6 h LPS, n = 4; 24 h LPS, n = 4–5; one-way ANOVA followed by Tukey multiple comparison tests; bars represent means ± SEM. * = *p* < 0.05, ** = *p* < 0.01.

It was expected that increased circulating HMGB1 may lead to reduced mRNA expression of its specific receptors, namely, RAGE and TLR4, and may also affect HMGB1 expression in the brain and periphery (spleen and liver). However, while their expression was not changed in the hypothalamus and spleen, LPS treatment significantly reduced HMGB1, RAGE and TLR4 expression in the liver (Figure 2), suggesting some feedback mechanism with its downregulated production and reduced receptor expression associated with increased plasma HMGB1 levels.

### 2.3. Systemic Septic-Like LPS-Induced Inflammation Led to Cytoplasmatic Translocation of Nuclear HMGB1 Immunoreactivity

Immunohistochemistry was used to analyze the localization of HMGB1 in the rat brain. In addition to the area postrema (AP), the median preoptic nucleus (MnPO) is known to play a pivotal role in fever induction pathways involving prostaglandin E2 action on EP3 receptors to stimulate heat generation and to inhibit heat dissipation pathways [40,45]. However, in contrast to the AP, the MnPO has a tight blood–brain barrier (BBB). Comparison of these two brain regions served to assess the importance of HMGB1 for the development of fever, inflammation in the brain and its potential relevance for immune-to-brain communication pathways, i.e., as a circulating (AP) or brain intrinsic mediator (MnPO). Specificity of staining was controlled using an antibody-specific blocking peptide or omitting the primary antibody (Supplementary Figure S1A,B). Indeed, HMGB1 immunoreactivity (IR) disappeared almost completely with some remaining background staining in these technical controls.

**Figure 3:**
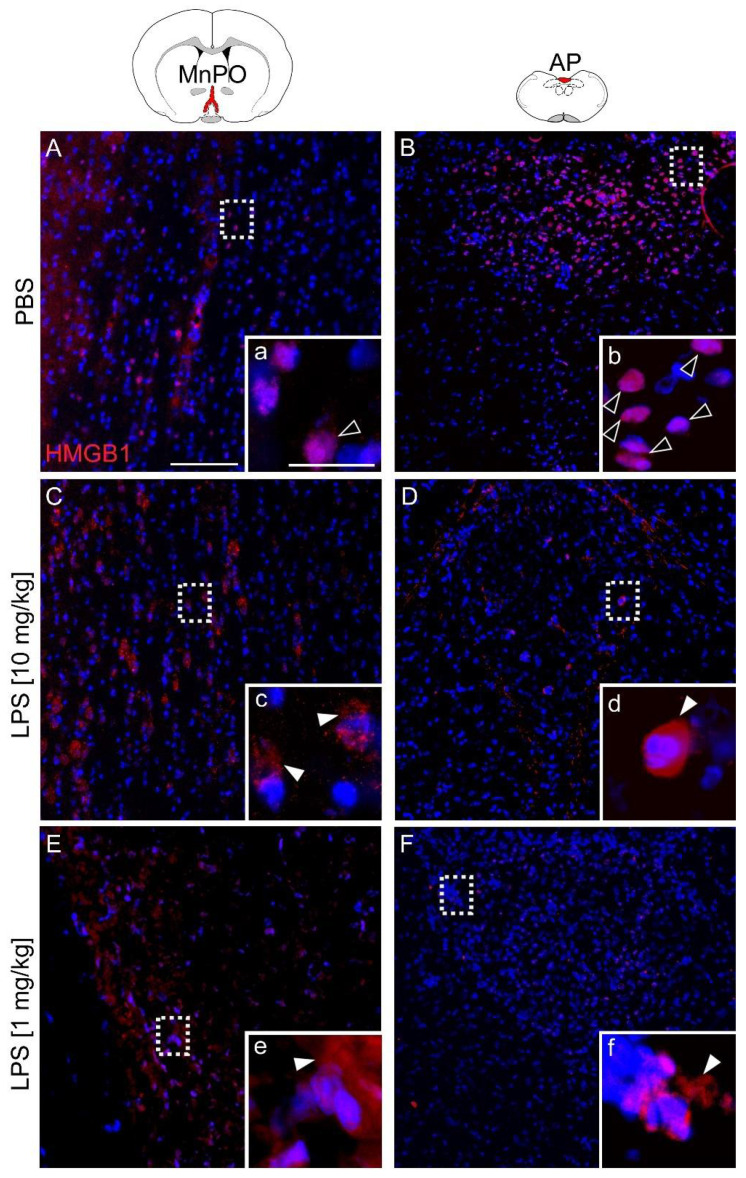
Immunohistochemical localization of HMGB1 expression (red) within the median preoptic nucleus (MnPO) and area postrema (AP) 24 h after injection of PBS or LPS. Rats treated with PBS showed intranuclear HMGB1 signals in both structures (**A**, a + **B**, b; open white arrowheads). After injection of LPS (10 mg/kg (**C**, c + **D**, d) or 1 mg/kg (**E**, e + **F**, f)) HMGB1 signals were detected perinuclearly within the cytoplasm (filled white arrowheads). Specific staining disappeared in technical controls (Supplementary Figure S1). The inserted boxes represent magnified images of marked areas (white dashed rectangles). Four mice per group of treatment (PBS, LPS 10 mg/kg, LPS 1 mg/kg) with at least two slides per animal were stained and representative pictures are displayed. Scale bars represent 100 µm (**A**) or 25 µm (a) and are applicable for all images.

In PBS controls, HMGB1-IR (red) was mainly detected as intranuclear (24 h, Figure 3A,B). In contrast, the HMGB1 signal was less condensed 24 h after LPS injection and was primarily observed as perinuclear and cytoplasmic staining (Figure 3C,D). In both, the AP (leaky BBB) and the MnPO (tight BBB) LPS treatment induced translocation of HMGB1-IR. Moreover, immunohistochemical analyses of the MnPO and AP in test sections from animals that received an injection of a lower LPS dose (1 mg/kg, 24 h) confirmed perinuclear translocation of HMGB1 during less severe (versus 10 mg/kg) LPS-induced inflammation (Figure 3E,F).

### 2.4. Disulfide-HMGB1 Significantly Increases IL-6 But Not TNFα Secretion in the Supernantans of Primary Neuro-Glial Cultures of the AP

**Figure 4:**
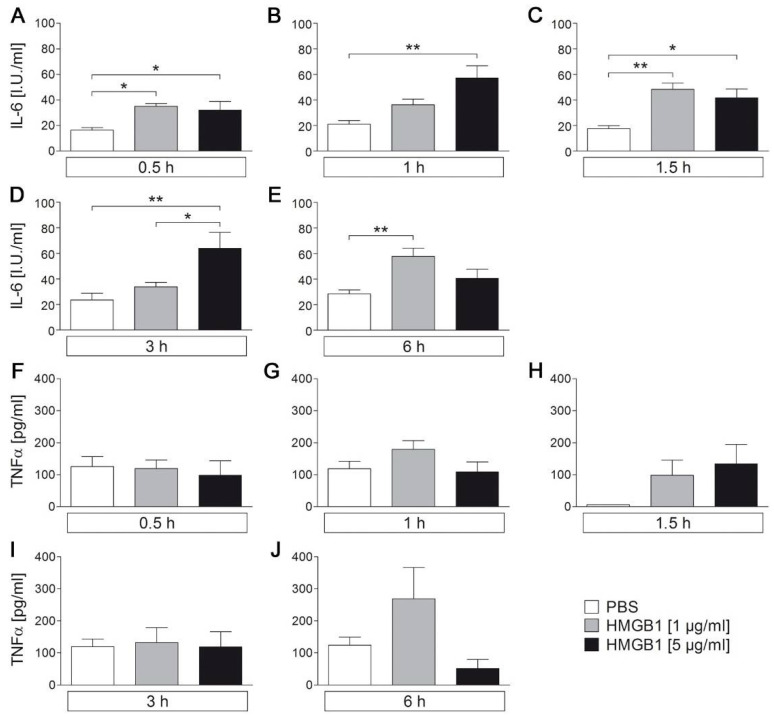
HMGB1-induced release of IL-6 and TNFα into supernatants of neuroglial primary cultures of the rat area postrema (AP). Primary cell cultures of the rat AP were used to characterize HMGB1-induced release of the pro-inflammatory cytokines IL-6 (**A**–**E**) and TNFα (**F**–**J**). AP cells were stimulated with HMGB1 (1 µg/mL or 5 µg/mL) for up to 6 h. While IL-6 release was enhanced due to stimulation with HMGB1, TNFα release was not affected. Bars represent means ± SEM. Numbers of investigated cultures in independent experiments are shown in Table 1. * = *p* < 0.05, ** = *p* < 0.01.

Recently, it has been shown that HMGB1 prolongs and enhances the febrile response to a low LPS dose (50 µg/kg) after turpentine pre treatment, suggesting a role for HMGB1 as a trigger of brain inflammation and as an endogenous pyrogen [32,33]. An intracerebroventricular (icv) injection of HMGB1 induced anorexia, with an increase in TNFα and IL-6 expression in the brain in mice [31] and fever with increased hypothalamic IL-1 levels in rats [32]. Here, sustained circulating and brain-intrinsically released HMGB1 levels may contribute to the long-lasting fever after injection of a septic-like LPS dose (10 mg/kg). Thus, HMGB1 should trigger a pro-inflammatory response in the AP, an important brain structure in immune-to-brain communication, with direct access not only to brain-derived but also circulating HMGB1. Indeed, stimulation of primary neuro-glial cultures of the AP with cytokine-like disulfide-HMGB1 significantly increased the concentration of IL-6 but not TNFα in supernatants of the primary cultures at 0.5 h, 1 h, 1.5 h, 3 h, and 6 h after HMGB1 stimulation (1 µg/mL, 5 µg/mL) compared to PBS control (Figure 4). The increase in IL-6 bioactivity was not, however, time or dose dependent. The number of independent experiments/cultures and cultured wells per treatment are listed in Table 1. Interestingly, we found that HMGB1 significantly increased levels of docosahexaenoic acid (DHA) in supernatants of AP cultures while arachidonic acid (AA) and eicosapentaenoic acid (EPA) levels were not altered suggesting some anti-inflammatory potential for released DHA (Supplementary Figure S2, 10 µg/mL HMGB1).

### 2.5. HMGB1-Induced Nuclear NFκB Translocation in Astrocytes and Microglia in Primary AP Cultures

**Figure 5:**
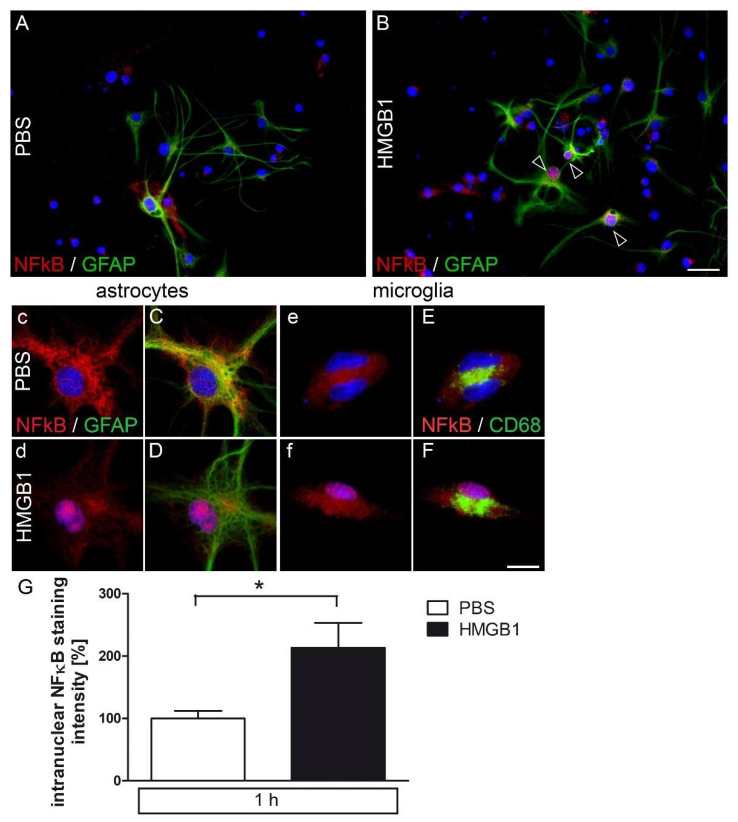
Immunocytochemical detection of NFκB signals in primary cell cultures of the rat area postrema (AP) after stimulation with HMGB1. Primary cell cultures of the AP were stimulated with HMGB1 (5 µg/mL) and after fixation used for immunocytochemistry. HMGB1-induced nuclear translocation of NFκB was observed in GFAP-positive astrocytes ((**A**–**D**), open white arrowheads) and CD68-positive activated microglia (**E**–**F**) after one hour of stimulation. Boxes with lower-case letters (c–f) show NFκB (red) and DAPI-stained nuclei (blue), while boxes with capitals show the overlay with respective cell type markers (green). Scale bars represent 25 µm (**A**,**B**) or 10 µm (**C**–**F**). Intranuclear intensity of NFκB staining (microglia and astrocytes) was quantified (**G**) from microphotographs out of three independent experiments (seven wells after HMGB1- and six wells after PBS stimulation). Data were normalized to mean PBS values set to 100%. Bars represent means ± SEM (*t*-test, * = *p* < 0.05).

HMGB1 activates RAGE/TLR4-NFκB signaling, for example, to induce IL-6 de novo synthesis [4]. Moreover, brain NFκB activation is known to be involved in the initiation of fever [46] and induction of sickness behavior [47]. In Figure 5A–G, nuclear NFκB-IR (p65) is shown one hour after HMGB1 stimulation (5 µg/mL) in glial fibrillary acidic protein (GFAP)-positive astrocytes (Figure 5C–D), and activated microglia (CD68, Figure 5E–F) compared to cytoplasmatic NFκB-IR in PBS-treated controls of primary AP cultures. For better visualization of NFκB staining (red) in astrocytes (GFAP) and microglia (CD68), microphotographs are shown with (upper case, Figure 5C–F) or without (lower case, Figure 5c–f) co-labelling of marker proteins (green). Overall, astrocytes more frequently showed HMGB1-induced nuclear NFκB translocation (Figure 5A,B). Evaluation of the mean nuclear staining intensity for NFκB confirmed significant activation of this pivotal signaling pathway by HMGB1 (Figure 5G). MAP2ab-labeled neurons did not show significant NFκB activation (data not shown). Notably, the pattern of NFκB staining in astrocytes and microglia showed some very robustly activated cells, as depicted in Figure 5A (PBS) versus 5B (HMGB1). Three out of several GFAP-positive astrocytes did clearly respond to HMGB1 stimulation, while nuclear NFκB staining did not increase in other astrocytes displayed (Figure 5B). Previous in vivo and in vitro studies have also shown LPS-induced nuclear NF-IL6 translocation in sCVOs of the rat brain with a peak between four and eight hours [48,49,50]. NF-IL6 has been proposed to play a role as an early pro- and late anti-inflammatory transcription factor during systemic inflammation [51]. However, HMGB1 stimulation did not induce a significant increase in the number of nuclear NF-IL6-IR, as percentage of all DAPI-stained nuclei, mainly found in non-neuronal cells compared to PBS controls (Supplementary Figure S3).

### 2.6. LPS-Stimuated AP Cultures Show a Decrease in Nuclear HMGB1-IR Compared to PBS Controls

**Figure 6:**
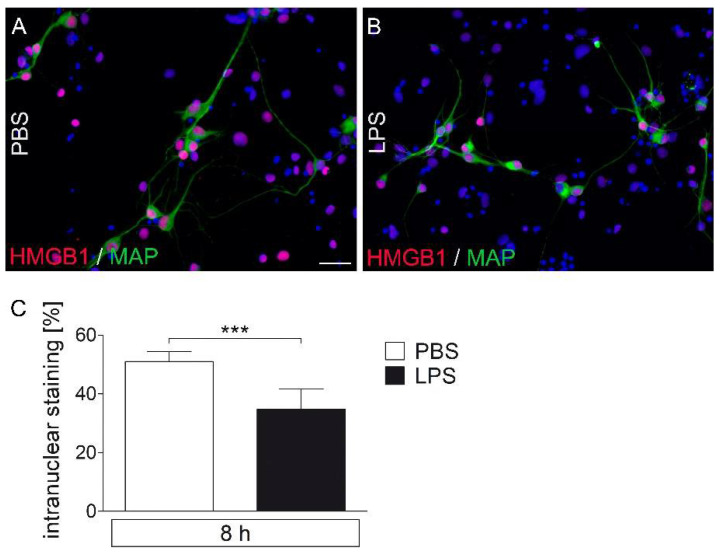
Immunocytochemical detection of HMGB1 signals in LPS-stimulated primary cell cultures of the rat area postrema (AP). After eight hours of stimulation with LPS (1 µg/mL), primary cell cultures were fixed and used for immunocytochemistry. HMGB1 signals (red) were detected in nuclei (blue) of MAP2ab-positive neurons (green) (**A**,**B**). The number of all HMGB1-positive nuclei was significantly attenuated after treatment with LPS (**C**). Six wells per group (LPS vs. PBS; 6–12 microphotographs per well) out of three independent experiments were analyzed. Scale bar represents 25 µm (**A**,**B**). Bars represent means ± SEM (*t*-test, *** = *p* < 0.0001) (**C**).

Primary AP cultures were stimulated for eight hours with LPS (1 μg/mL) or were treated with PBS as a control (Figure 6). Qualitatively, the number and intensity of intranuclear signals observed in PBS treated cultures (Figure 6A) decreased after LPS treatment (Figure 6B) as shown for MAP2ab-stained neuronal HMGB1 immunoreactivity. Analyses of the percentage of intranuclear signals confirmed a significant reduction in the percentage of cells with intranuclear HMGB1-IR with LPS compared to with PBS (Figure 6C), supporting a release of HMGB1 into the supernatants, as suggested for in vivo experiments during systemic septic-like LPS-induced inflammation using HMGB1-stained sections (Figure 3). Under control conditions, most of the detected MAP2ab-labeled neurons showed nuclear HMGB1 signals. Eight hours after LPS incubation, both HMGB1-positive and HMGB1-negative neurons were observed.

### 2.7. Dose–Response Experiments for the Rapid, Direct Cell Response to Stimulation with the TLR agonists HMGB1 and LPS Using Ca^2+^ imaging Revealed Only Very Few Responive Cells to HMGB1 Compared to LPS

**Figure 7:**
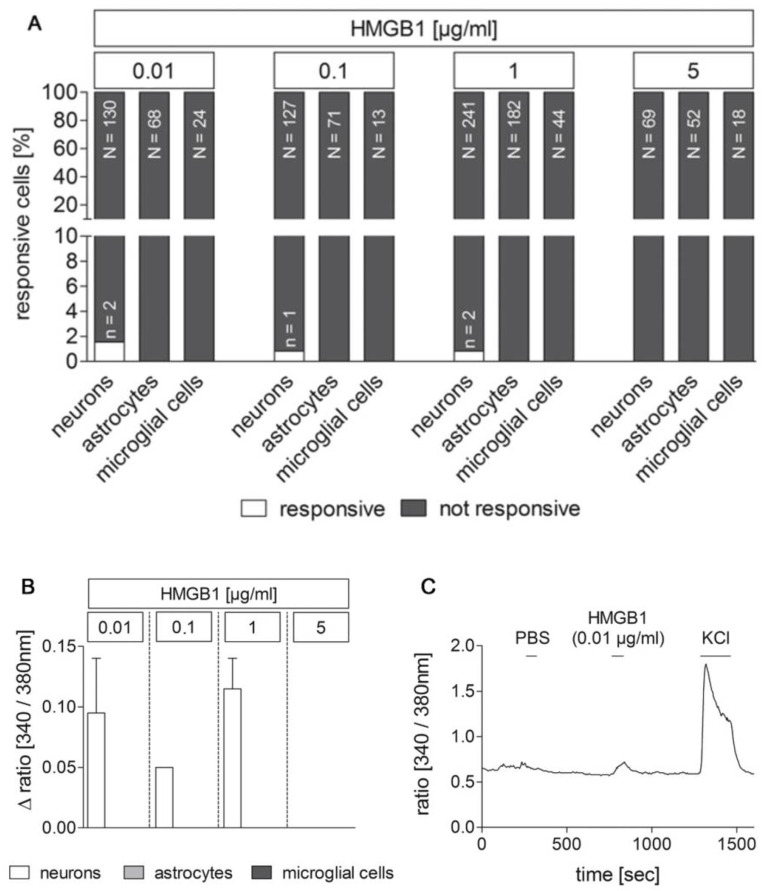
Responses of cultured area postrema (AP) cells upon stimulation with HMGB1 in Ca^2+^ imaging experiments. Primary cell cultures of the rat AP were used for Ca^2+^ imaging experiments 4–5 days after preparation. HMGB1 was applied in serial dilutions (0.01, 0.1, 1, 5 µg/mL) to identify stimulus-induced changes in the ratio [340/380 nm] indicative for changes in intracellular calcium concentrations [Ca^2+^]_i_. Cell types were characterized by immunocytochemistry after each experiment. A rather small population of neurons responded to stimulation with low doses of HMGB1 with an increase in [Ca^2+^]_i_, while astrocytes and microglial cells did not show any response ((**A**); n = responsive cells, N = total amount of investigated cells). Mean Δratios [340/380 nm] ± SEM of responsive cells are shown in (**B**). A representative example of an HMGB1 responsive cell is presented in C. PBS was applied as control, while KCl (50 mM) was used to confirm neuronal viability. The numbers of investigated cultures and independent experiments are provided in Table 2.

The investigation of the fast, direct cell responses to an application of the TLR agonists LPS and HMGB1 was carried out by analyzing the intracellular calcium concentration ([Ca^2+^]_i_) with Ca^2+^ imaging” (Figure 7, Supplementary Figure S4) as previously shown [42]. An increased [Ca^2+^]_i_ caused an increase in the ratio [340 nm/380 nm] and was assessed as cell activation (Δ ratio ≥ 0.05). Only cells, which did not react to the bolus application of PBS with a ratio change ≥ 0.05 and whose cell type was clearly identified by immunocytochemistry, were included in the analyses. Overall, hardly any cell showed a response to the application of PBS. Superperfusion with potassium chloride (KCl) served as a vitality control. The number of wells and independent cultures analyzed are given in Table 2.

Exposure to HMGB1 was investigated at serial dilutions of 0.01 µg/mL, 0.1 µg/mL, 1 µg/mL, and 5 µg/mL. HMGB1-application resulted in an increase in [Ca^2+^]_i_ in only 0.48% of the cells examined (Figure 7). Astrocytes and microglial cells were not responsive at any of the administered concentrations. In contrast, 1.54% of the neurons exposed to an HMGB1 concentration of 0.01 µg/mL reacted to this application. In each case, 0.83% of the neurons examined were responsive to the bolus application of HMGB1 at a concentration 0.1 µg/mL and 1 µg/mL. A dose effect was, therefore, not detectable. The level of induced changes in ratios of responsive neurons was relatively inhomogeneous and ranged from 0.05 to 0.14 (Figure 7B). An exemplary response of a neuron is presented in Figure 7C showing the absence of a ratio change due to the bolus application of PBS and an increase in the ratio [340 nm/380 nm] associated with the bolus application of HMGB1 (0.01 µg/mL). The superperfusion with KCl led to an increase in [Ca^2+^]_i_, which is typical for neurons (ratio [340 nm/380 nm]).

Moreover, LPS concentrations of 0.01 µg/mL, 0.1 µg/mL, 5 µg/mL, and 10 µg/mL were used in addition to 1 µg/mL to assess which LPS dose is best suited for the comparison with the response to HMGB1. A total of 3.67% of the cells examined were responsive (Supplementary Figure S4). All three cell types investigated (neurons, astrocytes, microglial cells) reacted to a bolus application of LPS at different concentrations. No dose dependency of the different cell types could be determined. 4.55% of the examined neurons and 3.85% of the examined astrocytes reacted to the bolus application of LPS at a concentration of 0.01 µg/mL. Only neurons stimulated with LPS at a concentration of 0.1 μg/mL were responsive, namely 1.96% of the neurons examined. The stimulation with LPS at a concentration of 1 µg/mL caused a ratio increase ≥0.05 in 6.09% of the neurons and in 2.68% of the astrocytes. 10% of the astrocytes and 7.69% of the microglial cells responded to the application of LPS with 5 µg/mL, and 6.52% of the neurons and 3.32% of the astrocytes to the application of LPS with 10 µg/mL similar to what has been previously shown [42]. Changes in the ratio of responsive cells ranged from 0.05–0.56. Overall, there was an inhomogeneous change in ratio, but astrocytes showed on average the highest increase in [Ca^2+^]_i_ and, thus, in the ratio [340 nm/380 nm] (Supplementary Figure S4B). The ratio measurement of an exemplary LPS-responsive astrocyte is shown in Supplementary Figure S4 with no change in the ratio to the PBS-bolus. The moderate increase in the ratio [340 nm/380 nm] in response to KCl superperfusion corresponds to the typical reaction of astrocytes.

Overall, we decided to choose the intermediate dose of 1 µg/mL for the following combined LPS/HMGB1 Ca^2+^ imaging experiments.

### 2.8. Previous LPS-Induced Priming Increased Ca^2+^ signal Responsiveness Following Single or Combined Stimulation with HMGB1

**Figure 8:**
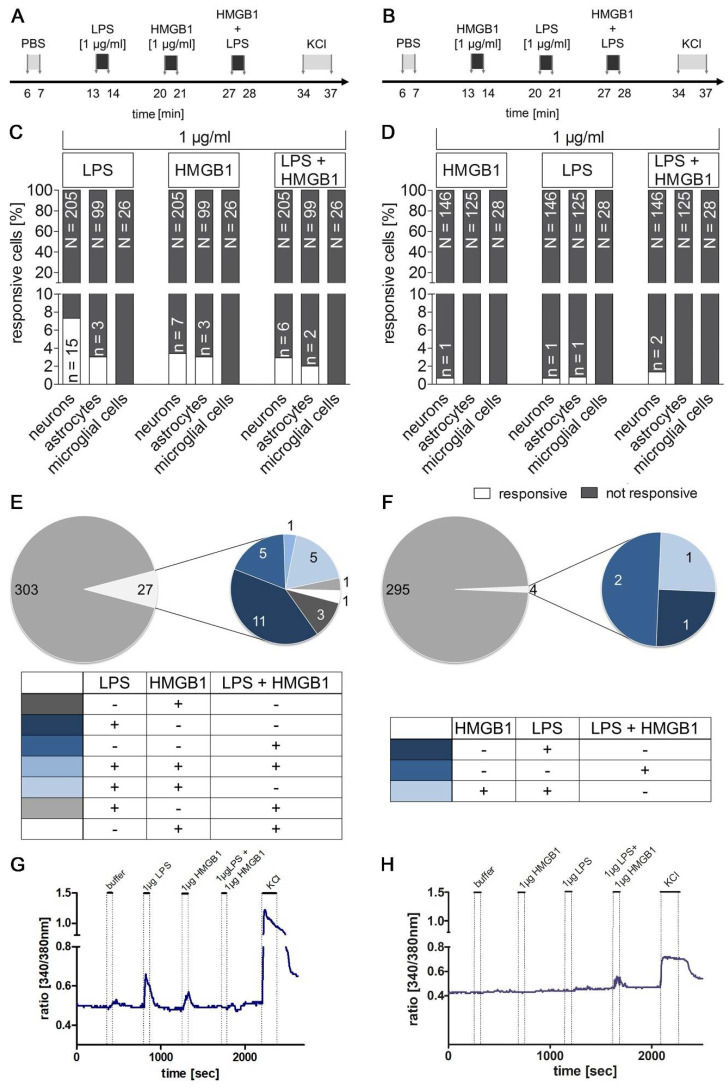
Stimulation with LPS primes cellular responses for following single or combined stimulation with HMGB1. Ca^2+^ imaging experiments were performed to test the effects of a first stimulation with LPS on responses upon following single or combined HMGB1 stimulation (**A**,**C**,**E**,**G**) and vice versa (**B**,**D**,**F**,**H**). Stimulation protocols are shown in (**A**) and (**B**). LPS was used as the first stimulus in (**A**); in (**B**) it was HMGB1. The percentage of responsive cells (n, white bars) out of all cells tested (N, grey) is visualized in (**C**,**D**). LPS pre treatment significantly increased the percentage of HMGB1-responsive cells (**E**) compared to (**F**). In contrast, the responsiveness to LPS was significantly reduced by the previous application of HMGB1 (**D**). The reaction pattern of responsive cells is summarized in (**E**,**F**), and representative examples of two responsive cells are presented in (**G**–**H**). Please note that some of the responsive cells reacted to several of the stimuli used with an increase in the intracellular calcium concentration. PBS was applied as control, while KCl (50 mM) was used to confirm neuronal viability. The numbers of investigated cultures and independent experiments are given in Table 3.

To test whether previous HMGB1 or LPS treatment can prime the responsiveness following single or combined stimuli, two combined protocols were applied (Figure 8A,B). A previous bolus application of LPS (Figure 8A) led to an increase in the percentage of responsive cells to HMGB1 with 3.4% of the neurons and 3% of the astrocytes (Figure 8C). Based on all cells and cell types examined a responsiveness of 3.03% to HMGB1 (1 µg/mL) was revealed compared with the dose–response experiments where only 0.43% of the cells examined were responsive (Figure 7). This observation suggests a seven-fold increase in responsive cells after LPS pre treatment (priming). Interestingly, after LPS-application astrocytes also showed Ca^2+^ signals to a bolus application of HMGB1 (1 µg/mL).

The inverse protocol was used to investigate the influence of HMGB1 on the LPS-induced reaction, i.e., HMGB1 followed by LPS alone and combined LPS/HMGB1 (Figure 8B). When HMGB1 was applied first, there was a nine-fold lower response of the cells to HMGB1 (3.03% in Figure 8C versus 0.33% in Figure 8D) compared to LPS pre treatment.

Figure 8E visualizes responsive cells according to their pattern of reactivity. Among 16 HMGB1-responsive cells, only three cells reacted to the bolus application of HMGB1. One cell reacted to all three bolus applications, namely LPS, HMGB1 and the combined application of LPS and HMGB1. Five cells responded to both LPS and HMGB1, and two cells responded to HMGB1 or LPS and the combined bolus application. In addition, there were only five cells that showed Ca^2+^ signals to the combined application of LPS and HMGB1. The percentage of LPS-responsive cells was similar to the dose-finding experiments (Supplementary Figure S4, 1 µg/mL).

Figure 8F depicts the response patterns to the inverse protocol with HMGB1 application as the first stimulus prior to an application of LPS. Only one neuron reacted to the application of HMGB1. This corresponds to 0.7% of the measured neurons and 0.33% of the total measured cells similar to what was found in the dose-finding experiment (Figure 7). However, the previous application of HMGB1 resulted in a strong reduction in the percentage of LPS-responsive cells. Thus, the percentage of LPS-responsive cells decreased with HMGB1 pre treatment compared to the inverse protocol (eight-fold, Figure 8A).

Two exemplary responses to both protocols are shown in Figure 8G,H. Overall, the present data suggest that LPS pre treatment primes brain cells in the AP to become more responsive to subsequent HMGB1 stimulation. Thus, in situations where there are high plasma LPS levels, like during sepsis, brain cells become more responsive to HMGB1. Indeed, the AP has direct access to circulating LPS and with some delay also to plasma and locally released HMGB1. This data supports an important role for HMGB1 as a mediator for immune-to-brain communication during septic-like systemic inflammation and may explain previously reported delayed and prolonged action of HMGB1 on the brain during sepsis. Vice versa, the presence of high plasma HMGB1 levels may reduce responsiveness to subsequent HMGB1 action on brain cells, potentially representing a protective mechanism.

## 3. Discussion

In the present study, we show that LPS-induced septic inflammation in rats is accompanied by late (24 h) and prolonged plasma HMGB1 levels, increased cytoplasmatic HMGB1 staining in the MnPO and AP and reduced nuclear HMGB-IR (AP) in primary neuro-glial AP-cell cultures. Our findings identify a potential role for locally released and circulating HMGB1 in mediating fever and neuroinflammation via these pivotal brain structures and fever induction during systemic septic inflammation. Indeed, stimulation of AP cultures with the cytokine-like disulfide-HMGB1 induced increased IL-6 bioactivity in supernatants, and NFκB activation primarily in astrocytes but also in microglia. Ca^2+^ imaging experiments further revealed evidence for a rather weak direct activation of neurons by disulfide-HMGB1. Stimulating the cultures with LPS before stimulating them with HMGB1 or combined HMGB1/LPS stimulation increased the numbers of HMGB1-responsive neurons seven-fold and also activated astrocytes. Administering HMGB1 before LPS stimulation, however, reduced LPS-induced Ca^2+^ signals. Overall, this is the first evidence that disulfide-HMGB1 is an endogenous humoral mediator in immune-to-brain communication via the AP. Its role in immune-to-brain communication seems to be enhanced by priming with naturally first occurring exogenous pathogens, such as LPS during systemic septic inflammation.

Circulating HMGB1 levels in the ng/mL range detected here are similar to those reported previously during turpentine- and LPS-induced inflammation in rats (2–6 ng/mL) [33], while even higher levels were detected in human plasma of septic patients (around 100–200 ng/mL) [22,52] and mice (~100–300 ng/mL) [22,25]. The circulating levels of HMGB1 can remain elevated for up to eight weeks in sepsis-surviving mice (>20 ng/mL) [26]. The concentrations of HMGB1 used in our cell culture experiments likely mimicked levels in the range that have been reported in clinical or animal studies in particular, as locally released HMGB1 will show even higher concentrations than in circulation.

The reduced HMGB1 expression in the liver observed in the current study is in accordance with a previous report showing reduced cell-type-specific expression also occurring in HMGB1-stimulated neutrophil granulocyte cultures from septic patients [53]. Sass et al. (2002) even reported a time-dependent reduction of HMGB1 expression in the liver with early increased (1–2 h) and late (6 h, 24 h) reduced levels during severe LPS-induced systemic inflammation (10 mg/kg) in mice [54]. Such downregulation of HMGB1 and its receptors RAGE and TLR4 may actually be related to some negative feedback in response to circulating HMGB1-acting on the liver.

Basal HMGB1 levels in the brain show a spatial distribution, in particular in the developing brain, and generally low levels in the adult brain [55]. However, brain HMGB1 translocates into the cytoplasm and occurs as a paracrine extracellular DAMP mediator during the cecal ligation and puncture model of sepsis [56], ischemia [57], seizure models [58], and hemorrhagic [59] or traumatic brain injury [60]. Interestingly, neutrophil granulocytes that are recruited to the brain during septic encephalopathy and other brain pathologies [61] may contribute to HMGB1 release during such brain pathologies [62,63]. HMGB1 is also known to be a common activator of neutrophil extracellular traps (NETs) and NET-induced thrombosis [64]. NETs are extracellular DNA fibers with associated antimicrobial peptides or granule proteins as e.g., myeloperoxidase that are released by activated neutrophils, and can bind and accumulate cytokines or infiltrating pathogens. Interactions between HMGB1 and TLR4 can enhance the formation of NETs, which have been shown to contribute to brain tissue damage [65] and provide a novel mechanism through which HMGB1 may contribute to the severity of neutrophil-associated inflammatory conditions [66]. Here, we found some preliminary evidence of extracellular traps stained by extracellular markers for DNA-histone complexes in the rat brain during severe septic like inflammation (Supplementary Figure S5). The formation of NETs was confirmed by immunofluorescence staining using a NET-specific marker: the combination of DNA-Histone1 complexes and H3Cit. As a reaction of NET formation, the host itself releases DNases to recycle NETs and thereby reduces the possible tissue-damaging effect of NETs [67]. In addition, DNase presence was confirmed in combination with positive signals for histone1-DNAcomplex formation [68]. However, the contribution of such phenomenon to the brain pathology in our model and its significance remains to be further investigated.

Findings from our study identify that a likely source for brain HMGB1 is the MnPO [45]. The locally released HMGB1 within the AP suggests a role in modulating important immune-to-brain communication signals in the sCVO with a leaky blood brain barrier. To this end, it has previously been demonstrated that the AP is a relay station for immune-to-brain communication sensing humoral mediators during systemic inflammatory insults [40,69], potentially including circulating HMGB1. Furthermore, we provide evidence for a cytokine-like action of disulfide-HMGB1 in the AP inducing IL-6 and NFκB activation, confirming previous in vivo and in vitro results of cytokine- and NFκB-induced activity of HMGB1 in the brain, at least for disulfide-HMGB1 [6,31,32,70,71]. While we did not investigate the redox form of locally released or circulating HMGB1, an oxidative milieu is found during severe systemic inflammatory insults [72]. Therefore, occurrence of the disulfide form for our in vivo experiments can be expected. In support of this notion, a previous report has shown that icv injection of fully reduced HMGB1 was partially oxidized to disulfide-HMGB1, inducing depressive-like symptoms via its inflammatory potential [73].

Notably, we did not detect any significant increase in TNFα release in our cell cultures of the AP by HMGB1, which was previously shown to be induced after icv injection of HMGB1 in the mouse brain [31,73]. This discrepancy may be related to differences in the experimental models (in vivo versus in vitro, rat versus mouse, AP versus other brain structures) or the absence of endothelial cells in our neuro-glial cultures. As such, human umbilical endothelial cells have been previously shown to induce TNFα with HMGB1 stimulation [74].

To further investigate the action of disulfide-HMGB1 in the AP we conducted Ca^2+^ imaging experiments for detection of direct and quick responses to this stimulus as previously shown for LPS and cytokines in this brain structure [42]. Using primary cultures of dorsal root ganglia, calcium transients were observed in two previous studies [75,76] where 7.55% of sensory neurons showed Ca^2+^ signals after disulfide-HMGB1 stimulation [76]. While Balosso et al. (2014) did not find any direct effect of disulfide-HMGB1 on Ca^2+^ signals, it appeared to be a potent enhancer of NMDA-induced responses [77]. Here, we did find a rather weak responsiveness of brain cells to HMGB1 for cultured neurons of the AP. However, pre-treating the AP cultures with LPS or stimulating them with a combination of HMGB1 and LPS induced a seven-fold increase in neuronal HMGB1 responsiveness, and astrocytes also became responsive. Interestingly, we did not detect any dose–response effects, either for HMGB1-induced cytokine release or for Ca^2+^ signal-induction. Similarly, Frank and colleagues reported a U-shaped response for cytokine-induction in microglia by disulfide-HMGB1 [6]. Thus, HMGB1 may rather have a dose-dependent permissive role than a major primary inflammatory role.

Previous studies revealed that HMGB1 can bind to cytokines, such as IL-1β and CpG oligodeoxynucleotides (CPG-ODN), a TLR-9 ligand, which enhanced its pro-inflammatory activity [78,79]. Such potential was also suggested for HMGB1 binding to LPS [78,80]. Indeed, HMGB1 has two binding sides for LPS and the combination enhances its proinflammatory effect for TNFα and IL-6-production in human peripheral blood monocytes [81,82] or mouse peritoneal macrophages in vitro [83]. However, Ivanov and colleagues (2007) confirmed that CpG-ODN co-stimulation with HMGB1 engaged TLR9 to elicit enhanced IL-6 production using bone marrow-derived dendritic cells, but such exaggerated responses were not observed for LPS and HMGB1 [84]. Here, we cannot rule out a potential contribution of direct interactions of LPS with HMGB1 to the observed priming response. Nonetheless, continuous superperfusion of cells with buffer between bolus stimulations should have ensured washout of the previous stimulus. Moreover, only eight of the 27 responsive cells, of the 303 cells tested, showed Ca^2+^ signals with combined LPS and HMGB1 bolus stimulation when the treatment protocol started with LPS, suggesting that direct interaction may not play a major role in eliciting a Ca^2+^-response.

The inverse treatment protocol sequence with HMGB1 as the first stimulus, revealed only four Ca^2+^ signals within 295 cells investigated, thus even reducing sensitivity to LPS eight-fold. Such a reduction may be related to the previously reported role of HMGB1 in mediating tolerance to repeated or previous LPS exposure. Li and colleagues (2013) used low dose (0.2 mg/kg) LPS pre-exposure to investigate tolerance effects to a subsequent septic (10 mg/kg) dose of LPS in mice [85]. Similar effects were also reported in vitro in primary cultures from dorsal root ganglia and the superficial dorsal horn of the spinal cord [86]. Under in vivo conditions, pre-exposure to LPS increased endogenous HMGB1 synthesis, and its inhibition by neutralizing antibodies enhanced, for example, circulating TNFα levels, reversing the suppression of inflammation noted with LPS tolerance [85]. Similar results were obtained by others in bone marrow-derived murine macrophages or after injection of 20 µg HMGB1 one hour before LPS stimulation (10 mg/kg) [87]. Recent studies, however, have shown opposite effects of enhanced LPS-induced IL-1β expression in microglia in vitro when pre-incubating with disulfide-HMGB1 for 4 h [6]. Moreover, injecting disulfide-HMGB1 into the cisterna magna 24 h before the LPS stimulus in vivo exaggerated the neuroinflammatory responses in mice [6]. In terms of our present results, we observed disulfide-HMGB1-induced tolerance to induce Ca^2+^ signals. To this end, such tolerance mechanisms may serve as a protective mechanism in various known pathologies that are accompanied by increased plasma HMGB1 levels, like pulmonary arterial hypertension [88], ankylosing spondylitis [89] or neurological disease that do not elicit overt sickness responses like fever. In these clinical scenarios HMGB1 may still serve as a biomarker for prediction and assessment of disease progression though [29].

In addition, likely deviations in inflammatory responsiveness may involve changes in receptor expression and function to LPS, which affect how immune cells respond to HMGB1. For example, our observation shows that astrocytes only responded to HMGB1 with pre-exposure to LPS. Indeed, TLR4 mediated action in astrocytes may contribute to HMGB1 action on the brain in experimental rat stroke models [21]. In addition, recent evidence in bone-marrow-derived macrophages revealed that constantly occurring cell surface TLR4 and RAGE trafficking between the cell surface and the cytoplasm is differentially altered by HMGB1 in an acute peripheral tissue trauma model [90]. Thus, these mechanisms may explain increasing evidence that HMGB1 acting during chronic stress [34], aging [37] or other types of sterile inflammation [33] primes second hit responses to infection and inflammation with exaggerated responses. Frank et al. (2015) suggested some contribution of the NLRP3 inflammasome in this response of HMGB1-induced priming [6]. Our present results, however, broaden such evidence for incidences where LPS increases plasma levels of HMGB1 that precede the inflammatory response in the brain, like during sepsis or obesity [91]. Subsequent HMGB1 occurrence in plasma and locally released by the brain acts, for example, as a late mediator of sepsis [19] or contributes to high fat diet-induced hippocampal inflammation i.e., obesity [92]. In conclusion, our data show that HMGB1 is an important humoral and brain inflammatory mediator in immune-to-brain communication with sCVOs like the AP representing important relay stations in this response, as previously reported for cytokines like IL-6 [93,94,95] or LPS [42,43,95].

It is important to note that HMGB1 is also known to have some protective proliferation and neurogenesis-promoting activities that should be taken into consideration, depending on the disease being investigated [96]. As such, icv HMGB1 treatment improved cognitive impairments and neurogenesis in a mouse model of Alzheimer’s disease [97]. However, conditional knockout of HMGB1 in a model of traumatic injury not only reduced contusion volume but even naive animals showed HMGB1-induced impaired spatial memory, suggesting a “double-edged sword” function for HMGB1 in the brain [98]. Along with the benefits of its inhibition, pitfalls should also be expected. Interestingly, we found that disulfide-HMGB1 increased the release of ω-3 unsaturated fatty acid DHA in primary neuro-glial cultures of the AP. DHA is known to have anti-inflammatory and health-promoting potential in the brain [99]. Therefore, such action of HMGB1 should be further investigated in future studies.

Some limitations of the current study should be highlighted. For example, we failed to quantify changes in IL-1β immunoreactivity after HMGB1 treatment in our primary neuro-glial cell cultures. One underlying mechanism for unchanged IL-1β immunoreactivity may pertain to the fact that such cultures show some basal activation status, most likely related to cell growth and division under non-stimulated conditions, which is reflected by our observation of basal activity of the transcription factors NFκB and NF-IL6 in these cultures. Moreover, only some brain cells showed HMGB1 reactivity like Ca^2+^ signals or NFκB activation, while others remained unaffected, confirming some modulatory role of HMGB1 on brain cell activity rather than being a strong stimulant. Previous studies have applied HMGB1 immunohistochemistry to assess changes in its appearance [100,101,102], as observed for the MnPO and the AP in the current study. However, quantitative measures were rather limited to more severe insults, like during subarachnoid hemorrhage [102]. Our current results suggest that HMGB1 immunohistochemistry may represent a qualitative, but not a quantitative, tool for tissue immunohistochemistry assessments with variations in cytoplasmatic and nuclear staining. While this was not the focus of the present manuscript, more quantitative analyses are warranted.

Overall, we provide further evidence for HMGB1 as a mediator of the latter stages of septic inflammation in the brain, likely acting via the MnPO and the sCVO AP. We further show that disulfide-HMGB1 holds all the necessary criteria as a humoral mediator for immune-to-brain communication with proposed action in the AP inducing genomic activation, cytokine production and a rather low capacity to induce neuronal Ca^2+^ signaling. Importantly, short-term pre-exposure to LPS seems to prime neurons and astrocytes to increase or induce their response to HMGB1. These observations may explain the importance of HMGB1 in mediating neuroinflammation during disease and sepsis.

## 4. Materials and Methods

### 4.1. Animals

Male Wistar rats with a body weight of 180–250 g were used for in vivo experiments. They were obtained from an in-house breeding colony, with parents descended from Charles River (Charles River Laboratories, Sulzfeld, Germany). Breeding, animal care and experimental procedures were performed according to the guidelines approved by the Hessian Ethical Committee (ethics approval number GI 18/2 No. 51/2008 (control sections for lower dose of LPS, 1 mg/kg) and GI 18/2 Nr. 1/2011). Rats were housed at a room temperature of 24 ± 1 °C and 50% humidity with artificial light from 7:00 AM to 7:00 PM.

For the duration of the experiment, animals were individually housed in cages located in a climate chamber (10′US/+5 to +40 DU, Weiss Umwelttechnik GmbH, Reiskirchen, Germany) with controlled ambient temperature of 28 ± 0.1 °C, 50% humidity and artificial light from 7 a.m. to 7 p.m. Eight days before the experiment, intra-abdominal radio transmitters (PTD-4000 E-Mitter/ER-40000 Receiver, Mini-Mitter Company Inc., Sunriver, USA) were implanted for stress-free measurement of locomotor activity and core body temperature. Pre- and post-surgery rats were treated with Meloxicam (2 mg/kg BW) (Metacam solution, 5 mg/mL, Boehringer Ingelheim Vetmedica GmbH, Ingelheim, Germany) for pain relief. Anesthesia was performed by intraperitoneal (i.p.) injection of a cocktail containing ketamine (50 mg/kg BW) (Ketamin 10%, Cp-pharma, Burgdorf, Germany), medetomidin (5 mg/kg BW) (Cepetor^®^, Pfizer Deutschland GmbH, Berlin, Germany) and acepromazine (0.5 mg/kg BW) (Vetranquil^®^ 1%, Albrecht GmbH, Aulendorf, Germany). Rats had open access to water and powdered standard lab chow (R-Z V1324-000, Ssniff Spezialdiäten GmbH, Soest, Germany). To record body temperature, an automatic data acquisition system was applied (VitalView 3.1, MiniMitter Co. Inc., Bend, OR, USA). For habituation to experimental procedures rats were daily handled at the same times at least five days before the experiment.

### 4.2. Treatment and Experimental Protocol

A total of 16 male rats were i.p. injected with LPS (10 mg/kg BW) (derived from Escherichia coli, serotype O111:B4, Sigma-Aldrich Chemie GmbH) diluted in sterile pyrogen-free phosphate buffered saline (PBS; Dulbecco’s Phosphate Buffered Saline, PAH GmbH, Pasching, Germany) or with an equal volume of PBS. All injections took place between 9.00 am and 10.40 am in 25-min intervals. Six (three animals with PBS, four with LPS) or twenty-four hours (four animals with PBS, five with LPS) after injection, rats were deeply anesthetized with an i.p. injection of sodium pentobarbital (60–100 mg/kg BW) (Narcoren, Merial, Halbermoos, Germany). Cardiac puncture was used to collect blood samples for later analysis of circulating cytokines. After that, transcardial perfusion with 300–400 mL of ice cold 0.9% saline was performed. Liver, spleen and brain were quickly removed and frozen in powdered dry ice. Until further analysis, samples were stored at −55 °C.

### 4.3. Tissue Processing

A cryostat (CryoStar NX50, Thermo Fisher Scientific, Dreieich, Germany) served to cut the brains in coronal 20 µm brain sections of specific brain structures including the median preoptic nucleus (MnPO) and the area postrema (AP). For identification of specific brain structures, the stereotactic rat brain atlas of Paxinos and Watson (1998) was used [103]. Sections were thaw-mounted on poly L-lysine-coated glass slides and stored at −55 °C until further processing. For PCR analysis, further brain sections were stacked on a glass slide to dissect the hypothalamus. Hypothalamus as well as liver and spleen samples were stored at −55 °C until RNA-extraction.

### 4.4. Immunohistochemistry

Frozen brain sections were set in the cryostat at −20 °C for ten minutes, air dried at room temperature for ten minutes, and fixed in 4% paraformaldehyde (PFA; Merck, Darmstadt, Germany) diluted in PBS (0.1 M) for ten minutes. Subsequently, sections were incubated with a solution used for permeabilization consisting of 0.5% Triton X-100 (Sigma-Aldrich Chemie GmbH) diluted in PBS (0.1 M). Slides were washed with 0.1% Tween^®^ 20 (Sigma-Aldrich Chemie GmbH, Steinheim am Albuch, Germany) in PBS (0.1 M) three times for five minutes each. Then, brain sections were incubated with a blocking solution containing 5% normal donkey serum (Biozol Diagnostica Vertrieb GmbG, Eching, Germany) and 5% bovine serum albumin (BSA; Sigma-Aldrich Chemie GmbH, Steinheim am Albuch, Germany) diluted in PBS (0.1 M) for 1 h at room temperature. After blocking, the solution was removed and slides were incubated with primary antibody (rabbit anti-HMGB1 1:100, abcam plc, Cambridge, UK) diluted in PBS containing 2.5% BSA for 24 h at 4 °C. Subsequently, unbound antibodies were removed, and slides were washed three times for ten minutes. Subsequently, slides were incubated with the secondary antibody (Cy3™ donkey anti-rabbit 1:500, Jackson Immuno Research Europe Ltd. Newmarked, UK) diluted in PBS (0.1 M) for 1 h. Excessive antibodies were removed by three washes for ten minutes with PBS. Sections were further stained with 4′,6-Diamidin-2-phenylindol (DAPI; MoBiTec, Göttingen, Germany) diluted 1:1000 in PBS (0.1 M) for ten minutes and slides were washed three times for five minutes each in PBS and cover slipped using Citifluor AF1 (Citifluor Ltd., London, UK).

Staining for extracellular trap marker proteins and DNase used for Supplementary Figure S5 was performed with a few differences, which are highlighted here: Brain sections were fixed in 2% paraformaldehyde (PFA; Merck, Darmstadt, Germany) diluted in PBS (0.1 M) for ten minutes. After washing, brain sections were incubated with a blocking solution containing 10% fetal calf serum (FCS, Sigma-Aldrich Chemie GmbH, Steinheim am Albuch, Germany) and 2% BSA (BSA; Sigma-Aldrich Chemie GmbH, Steinheim am Albuch, Germany) diluted in PBS (0.1 M) for 1 h at room temperature. Primary antibodies were mouse monoclonal IgG2a anti DNA/Histone (Millipore MAB3864, Billerica, MA, USA, 0.55 mg/mL; 1:100) combined with rabbit anti H3Cit (abcam plc, Cambridge, UK, ab5103, 0.79 mg/mL; 1:25) or combined with rabbit anti DNase1 (Invitrogen, Carlsbad, CA, USA, PA5-22017, 0.93 mg/mL; 1:100) diluted in blocking solution incubated for 24 h at 4 °C. Secondary antibodies (Alexa plus 488 goat anti-mouse IgG, A32723 Invitrogen, 1:500 and Alexa 633 goat anti-rabbit IgG, A21070, Thermo Scientific, Stock: 2 mg/mL, 1:500) were also diluted in blocking buffer and incubated for 2 h. Sections were further counterstained with DAPI (MoBiTec, Göttingen, Germany) and covered with ProLong^TM^ Gold Antifade Mountant (Invitrogen, P36930, Carlsbad, CA, USA).

### 4.5. Real-Time PCR

Trizol (Thermo Fisher Scientific Inc., Dreieich, Germany) was used to extract total RNA of hypothalamic sections, liver and spleen samples according to the manufacturer’s protocol. Reverse transcription of 1 µg of total RNA was performed by using 50 U moloney murine leukemia virus reverse transcriptase (M-MLV Reverse Transcriptase, Thermo Fisher Scientific), 50 µM random hexamers (Thermo Fisher Scientific Inc., Dreieich, Germany) and 10 mM dNTP mix (Sigma-Aldrich Chemie GmbH, Steinheim am Albuch, Germany) in a total reaction volume of 20 µL. Quantitative real-time PCR was carried out in duplicate with preoptimized primer/probe mixture and TaqMan universal PCR master mix (StepOnePlus Real-Time PCR, TaqMan Gene Expression Master Mix, Thermo Fisher Scientific Inc., Dreieich, Germany) on a StepOnePlus Real-Time PCR System (Applied Biosystems, Foster City, CA, USA). The housekeeping gene β-actin (Rn00667869_m1, Thermo Fisher Scientific Inc., Dreieich, Germany) was applied as a reference to normalize cDNA quantities between different samples. A control sample within a control group of the experiment was determined as the value of 1 and the sample values represent x-fold differences from this control sample (ΔΔCT-method). Assay IDs of the measured genes were as follows and all genes were purchased from Thermo Fisher Scientific Inc.: HMGB1 (Rn00566331_m1), RAGE (Rn00584249_m1), TLR2 (Rn02133647_s1) and TLR4 (Rn00569848_m1).

### 4.6. HMGB1 ELISA

HMGB1 plasma levels were measured with a rat specific enzyme-linked immunosorbent assay (ELISA; ST51011, IBL International GmbH, Hamburg, Germany) according to the manufacturer’s protocol. The detection limit was 0.313–10 ng/mL.

### 4.7. Preparation and Cultivation of AP Primary Cell Cultures

Wistar rat pups (4–6 days) obtained from the in-house breeding colony were used for all in vitro experiments. Rats of both sexes were decapitated with an age of 4–6 days and heads were immediately immersed in cold 70% ethanol. After removing the skull, the hindbrain and the connected cerebellum were transferred into petri dishes filled with cold and oxygenated Gey’s Balanced Salt Solution (GBSS; Sigma-Aldrich Chemie GmbH, Munich, Germany) containing 0.5% d-glucose (Sigma-Aldrich Chemie GmbH, Steinheim am Albuch, Germany). The area postrema (AP) is connected to the dorsal medulla surface and was isolated by using eye scissors under a binocular microscope. The isolated AP tissue samples of 3–5 pup rats were collected in petri dishes containing cold and oxygenated Hanks Balanced Salt Solution (HBSS: without Ca^2+^ and Mg^2+^, Biochrom GmbH, Berlin, Germany) supplemented with 20 mM HEPES (Sigma-Aldrich Chemie GmbH) and Penicillin-Streptomycin (Pen/strep, Thermo Fisher Scientific Inc., Waltham, MA, USA). Dispase I (2.4 U/mL) (Roche Diagnostics GmbH, Mannheim, Germany) solution in prepared HBBS containing HEPEs was used for enzymatic digestion for 1 h. The enzyme was inactivated by washing with EDTA (1 mM) (Sigma-Aldrich Chemie GmbH) in prepared HBBS and then transferred to neurobasal A (Life Technologies GmbH, Darmstadt, Germany) supplemented with penicillin (100 U/mL)/streptomycin (0.1 mg/mL), 2% B27 (Thermo Fisher Scientific Inc., Dreieich, Germany) and 2 mM 1-glutamine (Thermo Fisher Scientific Inc., Dreieich, Germany). After a mechanical dissociation, cells were counted with a Neubauer hemocytometer and diluted to a suspension at 50,000 cells/mL. For liquid chromatography–mass spectrometry analysis, a suspension of 100,000 cells/mL was used. Cells were plated onto a pre-warmed poly L-Lysine coated cover slip (15 × 15 mm) (Menzel GmbH, Braunschweig, Germany) forming the bottom of micro cultures of Flexiperm-micro 12-well (Greiner-Bio One GmbH, Frickenhausen, Germany) plates. Cultures were incubated in a humidified atmosphere of 5% CO_2_ and 95% air at 37 °C. To remove cellular debris, the medium was changed the next day and, depending on the experiment, also the fourth, fifth and sixth day.

### 4.8. Measurement of Intracellular Calcium Concentration

At 4–5 days after preparation, cells were loaded with Fura-2AM (1 µM in DMSO, Thermo Fisher Scientific Inc.) in full medium for 45 min in humidified atmosphere with 5% CO_2_ and 95% air at 37 °C. The coverslips were clamped under an inverse microscope (IMT-2, Olympus-Optical, Hamburg, Germany) in a chamber constructed in-house to be superperfused by a buffer containing 5 mM HEPES, 130 mM NaCl (Sigma-Aldrich Chemie GmbH, Steinheim am Albuch, Germany), 5mM KCl (Sigma-Aldrich Chemie GmbH, Steinheim am Albuch, Germany), 1.0 mM MgCl2 (Sigma Aldrich Chemie GmbH), 1.25 mM CaCl_2_ (Merck KGaA, Darmstadt, Germnay) and 10 mM d-glucose (Sigma-Aldrich Chemie GmbH, Steinheim am Albuch, Germany), at pH 7.4 with a flow rate of 2 mL/min and 37 °C. A filter-wheel-based excitation system was used to perform fluorescence measurements of 340 versus 380 nm, which was analyzed with MetaFluor 7.7.8.0 software (Visitron GmbH, Puchheim, Germany). Regions of interest were defined for single cells by a continuously variable diaphragm. The time course of emitted fluorescence (>515 nm) after alternating excitations at 340 nm and 380 nm, respectively, was recorded at 0.2–2 Hz using a Spot Pursuit digital CCD-camera (Model 23.0, Visitron GmbH). The 340/380 nm ratio is proportional to intracellular calcium concentration because of shorter excitation wavelength upon binding to calcium. These ratios were recorded and transferred to Excel (Windows Microsoft, Munich, Germany) for further analysis. The superfusion pump (miniplus-3; Ambimed Analysentechnik, Langenfels, Germany) was stopped for 60 s, and 100 µL of different drugs were applied into the chamber by bolus injection. Then, cells were superperfused with buffer for 6 min before the next drug injection. A control bolus application was performed with PBS and at last with potassium chloride (KCl, Sigma Aldrich Chemie GmbH), which was used as vitality test.

Cells were fixed with 4% freshly prepared PFA in PBS for following phenotypic identification by immune-labelling with polyclonal antisera or monoclonal antibodies against cell-specific marker proteins like microtubule associated protein (MAP)2ab (mouse AP-20 anti-MAP2ab; Sigma-Aldrich Chemie GmbH) for neurons, glial fibrillary acidic protein (rabbit anti-GFAP; DakoCytomation, Eching, Germany) for astrocytes and CD68 (mouse anti-rat CD68; Bio-Rad Laboratories, Puchheim, Germany) for microglial cells.

### 4.9. Stimulation of Primary AP Cultures for Further Analysis

After 6 days of cultivation, cells were incubated with disulfide HMGB1 (HMGBiotech S.r.l., Milan, Italy; 1 µg/mL or 5 µg/mL) or PBS in full medium for 0.5 h, 1 h, 1.5 h, 3 h or 6 h in a humidified atmosphere of 5% CO_2_, and 95% air at 37 °C. Supernatants were removed and stored at −24 °C for cytokine measurements. For subsequent immunocytochemistry, cells were fixed for 15 min with 4% PFA (Sigma-Aldrich Chemie GmbH).

### 4.10. Cytokine Measurements with Bioassays

TNFα was quantified due to the cytotoxic effects on the mouse fibrosarcoma cell line WEHI 164 subclone 13 and a murine TNFα standard (National Institute for Biological Standards and Control, Potters Bar, UK). The quantification of IL-6 was achieved by its proliferative effect on B9 hybridoma cell line and a human IL-6 standard (National Institute for Biological Standards and Control, Potters Bar, UK). An influence on the used bioassays by other components present in the supernatants or plasma samples can be excluded by the application of a neutralizing TNF binding protein or a neutralizing antiserum against rat IL-6, as previously shown [104]. The detection limits were 3 IU/mL for IL-6 and 6.0 pg/mL for TNF-α. For further details on both assays, see [95].

### 4.11. Immunocytochemical Characterization of NFκB, NF-IL6 and IL-1β Expression

Cells were washed with PBS three times for five minutes, followed by a two-hour incubation at room temperature with blocking solution containing 10% fetal calf serum (PAA GmbH, Pasching, Austria) diluted in PBS with 0.05% Triton X-100. Primary antibodies (mouse anti-rat CD68 1:1000; mouse anti-GFAP 1:800, Merck Chemicals GmbH, Darmstadt, Germany; rabbit anti-GFAP 1:800; goat anti-rat IL-1β (1:1000) R&D Systems, Minneapolis, USA; mouse anti-MAP2ab 1:600; rabbit anti-NFκB 1:2000 Santa Cruz Biotechnology, Santa Cruz, USA; rabbit anti-NF-IL6 1:5000, Santa Cruz Biotechnology) were diluted in blocking solution and incubated with the cells for 24 h at room temperature in a humidified chamber or for 48 h at 4 °C. Three washes for five minutes with PBS containing 0.05% Triton X-100 (PBS-T) were used to remove unbound antibodies. Thereafter, cells were incubated with secondary antibodies such as Alexa488 donkey anti-mouse (1:500, Thermo Fisher Scientific Inc.), Alexa488 donkey anti-rabbit (1:500, Thermo Fisher Scientific Inc.), Cy3™ donkey anti-goat (1:500, Jackson Immuno Research Europe Ltd., Newmarket, UK), Cy3™ donkey anti-rabbit (1:800, Jackson Immuno Research Europe Ltd.) or Cy3™ goat anti-mouse (1:800, Jackson Immuno Research Europe Ltd.) diluted in blocking solution for 2 h at room temperature. After unbound secondary antibodies were removed by washing with PBS-T three times for five minutes each, cells were incubated with DAPI for ten minutes for nuclear staining. Subsequently, DAPI was removed and cells were washed with PBS-T three times for five minutes each before the coverslips were embedded using Citifluor AF1.

### 4.12. Microscopic Analysis

An Olympus BX50 epifluorescence microscope (Olympus Optical, Hamburg, Germany) was used to examine stained brain sections and cell cultures. Pictures were taken with a black-and-white Spot Insight camera (Diagnostic Instruments, Visitron Systems, Puchheim, Germany). Counts of intracellular HMGB1- and NFIL-6 staining were carried out on photomicrographs made with a 20-fold magnification. For this purpose, the cells were subdivided into five areas using a grit. Within these areas, a defined region with similar cell density was examined. In this way, five photomicrographs were taken and HMGB1/NFIL-6 positive nuclei counted as percentage of nuclei (DAPI) for each well.

Intranuclear intensity of NFκB staining was quantified for microglia and astrocytes from microphotographs out of three independent experiments (seven wells after HMGB1- and six wells after PBS stimulation). For this purpose, the outline of 92 GFAP- or CD68 co-labelled nuclei (DAPI, blue) were used as active region of interest to quantify NFκB immunoreactivity (red channel) using the Metamorph software (Molecular Devices, San Jose, CA, USA). Data were normalized to mean PBS values, set to 100% for astrocytes and microglia and represent the mean of each well of the mean of each group (HMGB1 vs. PBS) to account for differences between the size and staining intensity between cell types.

For Supplementary Figure S5, the samples were examined microscopically on a Leica TCS SP5 AOBS confocal inverted-base fluorescence microscope with a HCX PL APO CS 10 × 0.40 dry and 40 × 0.75–1.25 oil immersion objective with an Argon, 405 nm and 633 nm laser. The settings were adjusted using isotype control antibodies in separate preparations.

### 4.13. Liquid Chromatography–Tandem Mass Spectrometry

Primary cell cultures of the area postrema were incubated with LPS (10 µg/mL), HMGB1 (10 µg/mL) or PBS for 48 h. All cell supernatants were removed after 48 h. Subsequently, each well was washed with 200 µL methanol (Carl Roth GmbH und Co. KG, Karlsruhe, Germany) and 5 µL butylhydroxytoluene (1 mM) (Sigma-Aldrich Chemie GmbH) and this solution was added to the supernatants. The supernatants and washing solutions of six equally treated wells (≈600,000 cells in total) were pooled for one sample. Three pooled samples for each treatment were stored at −80 °C until further analysis. Lipid mediators and their precursors (fatty acids) were extracted by using solid phase extraction columns (Strata X polymeric reversed phase, 100 mg/3 mL). Columns were equilibrated with 3.5 mL methanol (100%) and then 3.5 mL water. To get a diluted sample solution with 10% methanol, 8700 µL water were added to each sample before samples were loaded onto the columns. Then, the columns were washed with 3.5 mL methanol (10%). After that, lipid mediators and corresponding fatty acids were eluted with 3 × 500 µL methanol (100%). Under a stream of nitrogen gas, methanol was evaporated. The dried extract was resuspended with 50 µL of acetonitrile (25%). 10 µL of resuspended extract were injected into a Dionex UltiMate 3000 UHPLC (Thermo Fisher Scientific). Lipid mediators and corresponding fatty acids were separated with a reversed phase Kinetex C18 2.6-µm column (100 × 2.1 mm, 100 Å) and a binary solvent system consisting of a mobile phase A (100% water with 0.1% formic acid) and mobile phase B (100% acetonitrile with 0.1% formic acid) with a flow rate of 500 µL/min. The gradient was 27–35% B in 15 min, 35–60% B in 2 min and 60–80% B in 10 min. For tandem mass spectrometry, a quadrupol orbital trapping mass spectrometer (Q Exactive, Thermo Fisher Scientific) was used at a mass resolution of 70,000 at m/z 200 (17,500 at m/z 200 in MS/MS mode) with electrospray ionization in negative-ion mode. The method used was composed of full-scan mass spectrometry (MS) and targeted tandem MS for lipid mediators with defined mass and retention time. Transitions of lipid mediators were selected by using previously published data [105,106]. Fatty acids AA, DHA and EPA were quantified from full-scan MS. Calibration curves were formed by plotting the area under the curve of fatty acid or of the transition of lipid mediators from extracted ion chromatogram against the concentrations of serial dilutions of the compound ranging from 1 ng/mL to 256 ng/mL or in case of fatty acids 100–25,600 ng/mL standard. Calculated concentrations of lipid mediators and fatty acids were adjusted based on recovery rates determined from quality control samples contained defined amounts of the analytes (10 ng/mL for lipid mediators, 1000 ng/mL for fatty acids) in matrix. Lipid mediators were not consistently detected and, thus, only results for fatty acids are given.

### 4.14. Data Analysis

Body core temperature was compared using a two-way repeated measures analysis of variance (ANOVA) with the between subject factor treatment and the within subject factor time at 15 min intervals. For this purpose, data were divided into intervals of 2 h and data analysis was followed by Bonferroni correction for multiple comparisons (Statistica 10, Stat Soft Europe GmbH, Hamburg, Germany). Percentages of intranuclear NFκB-, HMGB1-, and NF-IL6 stainings were compared as mean of the mean with a t-test (Prism 5 software, Graph Pad, San Diego, CA, USA). For mRNA expression, fatty acids, and cytokine data, one-way ANOVA was applied followed by Tukey multiple comparison tests (Prism 5 software, Graph Pad, San Diego, CA, USA). PBS-treated animals from 6- and 24-h time points were pooled, as no difference was detected between these control groups for cytokine and mRNA expression analyses. Ca^2+^ imaging data are presented as descriptive data. Statistical significance was considered to be *p* < 0.05. Means ± SEM are shown for all data presented.

## Figures and Tables

**Table 1:** Number of wells incubated per treatment and duration of stimulation as well as the number of independent culture experiments.

Treatment	Time	Number of Wells	Number of Independent Cultures
PBS	0.5 h	7	5
	1 h	6	4
	1.5 h	6	5
	3 h	7	5
	6 h	7	5
HMGB1 (1 µg/mL)	0.5 h	7	4
	1 h	8	4
	1.5 h	6	4
	3 h	7	4
	6 h	7	4
HMGB1 (5 µg/mL)	0.5 h	5	2
	1 h	5	3
	1.5 h	6	3
	3 h	5	3
	6 h	6	3

**Table 2:** Number of wells and independent experiments for the Ca^2+^ imaging experiments with HMGB1.

HMGB1	Number of Wells	Number of Independent Cultures
0.01 µg/mL	8	5
0.1 µg/mL	12	6
1 µg/mL	21	13
5 µg/mL	6	3

**Table 3:** Number of wells and independent experiments for the Ca^2+^ imaging experiments with combined protocols.

Protocol	Number of Wells	Number of Independent Cultures
LPS → HMGB1 → LPS + HMGB1	14	8
HMGB1 → LPS → LPS + HMGB1	15	9

## Data Availability

The data presented in this study are available on reasonable request from the corresponding author.

## Supplementary Materials

The following are available online at https://www.mdpi.com/article/10.3390/ph14060558/s1, Supplementary Figure S1: Technical controls for immunohistochemical localization of HMGB1 within the median preoptic nucleus (A, MnPO) and area postrema (B, AP). Supplementary Figure S2: Stimulation with HMGB1 increases the release of docosahexaenoic acid (DHA) from cultures of the area postrema (AP), Supplementary Figure S3: HMGB1 treatment did not significantly alter NF-IL6 activation in primary cultures of the area postrema (AP), Supplementary Figure S4: Responses of cultured area postrema (AP) cells upon stimulation with LPS in Ca^2+^ imaging experiments, Supplementary Figure S5: Immunofluorescence staining of extracellular trap- markers and DNase in the area postrema (AP) 8 h after LPS stimulation, Supplementary Table S1: Number of wells and independent experiments for the Ca^2+^ imaging experiments with LPS.

## References

[1] Catez F., Yang H., Tracey K.J., Reeves R., Misteli T., Bustin M. (2004). Network of dynamic interactions between histone H1 and high-mobility-group proteins in chromatin. Mol. Cell. Biol..

[2] Ueda T., Chou H., Kawase T., Shirakawa H., Yoshida M. (2004). Acidic C-tail of HMGB1 is required for its target binding to nucleosome linker DNA and transcription stimulation. Biochemistry.

[3] Kang R., Chen R., Zhang Q., Hou W., Wu S., Cao L., Huang J., Yu Y., Fan X.G., Yan Z. (2014). HMGB1 in health and disease. Mol. Aspects Med..

[4] Lotze M.T., Tracey K.J. (2005). High-mobility group box 1 protein (HMGB1): Nuclear weapon in the immune arsenal. Nat. Rev. Immunol..

[5] Bianchi M.E. (2007). DAMPs, PAMPs and alarmins: All we need to know about danger. J. Leukoc. Biol..

[6] Frank M.G., Weber M.D., Fonken L.K., Hershman S.A., Watkins L.R., Maier S.F. (2016). The redox state of the alarmin HMGB1 is a pivotal factor in neuroinflammatory and microglial priming: A role for the NLRP3 inflammasome. Brain Behav. Immun..

[7] Faraco G., Fossati S., Bianchi M.E., Patrone M., Pedrazzi M., Sparatore B., Moroni F., Chiarugi A. (2007). High mobility group box 1 protein is released by neural cells upon different stresses and worsens ischemic neurodegeneration in vitro and in vivo. J. Neurochem..

[8] Shi Y., Zhang L., Teng J., Miao W. (2018). HMGB1 mediates microglia activation via the TLR4/NF-kappaB pathway in coriaria lactone induced epilepsy. Mol. Med. Rep..

[9] Qiu J., Nishimura M., Wang Y., Sims J.R., Qiu S., Savitz S.I., Salomone S., Moskowitz M.A. (2008). Early release of HMGB-1 from neurons after the onset of brain ischemia. J. Cereb. Blood Flow Metab..

[10] Peek V., Neumann E., Inoue T., Koenig S., Pflieger F.J., Gerstberger R., Roth J., Matsumura K., Rummel C. (2020). Age-Dependent Changes of Adipokine and Cytokine Secretion From Rat Adipose Tissue by Endogenous and Exogenous Toll-Like Receptor Agonists. Front. Immunol..

[11] Antoine D.J., Harris H.E., Andersson U., Tracey K.J., Bianchi M.E. (2014). A systematic nomenclature for the redox states of high mobility group box (HMGB) proteins. Mol. Med..

[12] Janko C., Filipovic M., Munoz L.E., Schorn C., Schett G., Ivanovic-Burmazovic I., Herrmann M. (2014). Redox modulation of HMGB1-related signaling. Antioxid. Redox Signal..

[13] Perry V.H., Cunningham C., Holmes C. (2007). Systemic infections and inflammation affect chronic neurodegeneration. Nat. Rev. Immunol..

[14] Denes A., Ferenczi S., Kovacs K.J. (2011). Systemic inflammatory challenges compromise survival after experimental stroke via augmenting brain inflammation, blood- brain barrier damage and brain oedema independently of infarct size. J. Neuroinflamm..

[15] Spencer S.J., Mouihate A., Pittman Q.J. (2007). Peripheral inflammation exacerbates damage after global ischemia independently of temperature and acute brain inflammation. Stroke.

[16] Schweighofer H., Rummel C., Roth J., Rosengarten B. (2016). Modulatory effects of vagal stimulation on neurophysiological parameters and the cellular immune response in the rat brain during systemic inflammation. Intensive Care Med. Exp..

[17] Tanaka M., Toldi J., Vecsei L. (2020). Exploring the Etiological Links behind Neurodegenerative Diseases: Inflammatory Cytokines and Bioactive Kynurenines. Int. J. Mol. Sci..

[18] Nishibori M., Mori S., Takahashi H.K. (2019). Anti-HMGB1 monoclonal antibody therapy for a wide range of CNS and PNS diseases. J. Pharmacol. Sci..

[19] Andersson U., Tracey K.J. (2011). HMGB1 is a therapeutic target for sterile inflammation and infection. Annu. Rev. Immunol..

[20] Kim I.D., Lee H., Kim S.W., Lee H.K., Choi J., Han P.L., Lee J.K. (2018). Alarmin HMGB1 induces systemic and brain inflammatory exacerbation in post-stroke infection rat model. Cell Death Dis..

[21] Famakin B.M., Tsymbalyuk O., Tsymbalyuk N., Ivanova S., Woo S.K., Kwon M.S., Gerzanich V., Simard J.M. (2020). HMGB1 is a Potential Mediator of Astrocytic TLR4 Signaling Activation following Acute and Chronic Focal Cerebral Ischemia. Neurol. Res. Int..

[22] Wang H., Bloom O., Zhang M., Vishnubhakat J.M., Ombrellino M., Che J., Frazier A., Yang H., Ivanova S., Borovikova L. (1999). HMG-1 as a late mediator of endotoxin lethality in mice. Science.

[23] Ren C., Tong Y.L., Li J.C., Dong N., Hao J.W., Zhang Q.H., Yao Y.M. (2017). Early antagonism of cerebral high mobility group box-1 protein is benefit for sepsis induced brain injury. Oncotarget.

[24] Wang H., Yang H., Tracey K.J. (2004). Extracellular role of HMGB1 in inflammation and sepsis. J. Intern. Med..

[25] Yang H., Ochani M., Li J., Qiang X., Tanovic M., Harris H.E., Susarla S.M., Ulloa L., Wang H., DiRaimo R. (2004). Reversing established sepsis with antagonists of endogenous high-mobility group box 1. Proc. Natl. Acad. Sci. USA.

[26] Chavan S.S., Huerta P.T., Robbiati S., Valdes-Ferrer S.I., Ochani M., Dancho M., Frankfurt M., Volpe B.T., Tracey K.J., Diamond B. (2012). HMGB1 mediates cognitive impairment in sepsis survivors. Mol. Med..

[27] Sunden-Cullberg J., Norrby-Teglund A., Rouhiainen A., Rauvala H., Herman G., Tracey K.J., Lee M.L., Andersson J., Tokics L., Treutiger C.J. (2005). Persistent elevation of high mobility group box-1 protein (HMGB1) in patients with severe sepsis and septic shock. Crit. Care Med..

[28] Parker T.M., Nguyen A.H., Rabang J.R., Patil A.A., Agrawal D.K. (2017). The danger zone: Systematic review of the role of HMGB1 danger signalling in traumatic brain injury. Brain INJ.

[29] Paudel Y.N., Shaikh M.F., Chakraborti A., Kumari Y., Aledo-Serrano A., Aleksovska K., Alvim M.K.M., Othman I. (2018). HMGB1: A Common Biomarker and Potential Target for TBI, Neuroinflammation, Epilepsy, and Cognitive Dysfunction. Front. Neurosci..

[30] Foo H., Ng K.P., Tan J., Lim L., Chander R.J., Yong T.T., Kandiah N. (2018). Interaction between APOE-varepsilon4 and HMGB1 is associated with widespread cortical thinning in mild cognitive impairment. J. Neurol. Neurosurg. Psychiatry.

[31] Agnello D., Wang H., Yang H., Tracey K.J., Ghezzi P. (2002). HMGB-1, a DNA-binding protein with cytokine activity, induces brain TNF and IL-6 production, and mediates anorexia and taste aversion. Cytokine.

[32] O’Connor K.A., Hansen M.K., Rachal Pugh C., Deak M.M., Biedenkapp J.C., Milligan E.D., Johnson J.D., Wang H., Maier S.F., Tracey K.J. (2003). Further characterization of high mobility group box 1 (HMGB1) as a proinflammatory cytokine: Central nervous system effects. Cytokine.

[33] Piotrowski J., Jedrzejewski T., Pawlikowska M., Wrotek S., Kozak W. (2019). High mobility group box 1 protein released in the course of aseptic necrosis of tissues sensitizes rats to pyrogenic effects of lipopolysaccharide. J. Therm. Biol..

[34] Frank M.G., Weber M.D., Watkins L.R., Maier S.F. (2015). Stress sounds the alarmin: The role of the danger-associated molecular pattern HMGB1 in stress-induced neuroinflammatory priming. Brain Behav. Immun..

[35] Wang B., Huang X., Pan X., Zhang T., Hou C., Su W.J., Liu L.L., Li J.M., Wang Y.X. (2020). Minocycline prevents the depressive-like behavior through inhibiting the release of HMGB1 from microglia and neurons. Brain Behav. Immun..

[36] Wang B., Lian Y.J., Su W.J., Liu L.L., Li J.M., Jiang C.L., Wang Y.X. (2019). FrHMGB1 and dsHMGB1 activate the kynurenine pathway via different mechanisms in association with depressivelike behavior. Mol. Med. Rep..

[37] Fonken L.K., Frank M.G., Kitt M.M., D’Angelo H.M., Norden D.M., Weber M.D., Barrientos R.M., Godbout J.P., Watkins L.R., Maier S.F. (2016). The Alarmin HMGB1 Mediates Age-Induced Neuroinflammatory Priming. J. Neurosci..

[38] Rummel C., Sachot C., Poole S., Luheshi G.N. (2006). Circulating interleukin-6 induces fever through a STAT3-linked activation of COX-2 in the brain. Am. J. Physiol. Regul. Integr. Comp. Physiol..

[39] Eskilsson A., Mirrasekhian E., Dufour S., Schwaninger M., Engblom D., Blomqvist A. (2014). Immune-Induced Fever Is Mediated by IL-6 Receptors on Brain Endothelial Cells Coupled to STAT3-Dependent Induction of Brain Endothelial Prostaglandin Synthesis. J. Neurosci..

[40] Roth J., Harre E.M., Rummel C., Gerstberger R., Hubschle T. (2004). Signaling the brain in systemic inflammation: Role of sensory circumventricular organs. Front. Biosci..

[41] Simm B., Ott D., Pollatzek E., Murgott J., Gerstberger R., Rummel C., Roth J. (2016). Effects of prostaglandin E2 on cells cultured from the rat organum vasculosum laminae terminalis and median preoptic nucleus. Neuroscience.

[42] Wuchert F., Ott D., Murgott J., Rafalzik S., Hitzel N., Roth J., Gerstberger R. (2008). Rat area postrema microglial cells act as sensors for the toll-like receptor-4 agonist lipopolysaccharide. J. Neuroimmunol..

[43] Wuchert F., Ott D., Rafalzik S., Roth J., Gerstberger R. (2009). Tumor necrosis factor-alpha, interleukin-1beta and nitric oxide induce calcium transients in distinct populations of cells cultured from the rat area postrema. J. Neuroimmunol..

[44] Roth J., Rummel C., Barth S.W., Gerstberger R., Hubschle T. (2009). Molecular aspects of fever and hyperthermia. Immunol. Allergy Clin. N. Am..

[45] Lazarus M., Yoshida K., Coppari R., Bass C.E., Mochizuki T., Lowell B.B., Saper C.B. (2007). EP3 prostaglandin receptors in the median preoptic nucleus are critical for fever responses. Nat. Neurosci..

[46] Rummel C., Gerstberger R., Roth J., Hubschle T. (2011). Parthenolide attenuates LPS-induced fever, circulating cytokines and markers of brain inflammation in rats. Cytokine.

[47] Nadjar A., Bluthe R.M., May M.J., Dantzer R., Parnet P. (2005). Inactivation of the cerebral NFkappaB pathway inhibits interleukin-1beta-induced sickness behavior and c-Fos expression in various brain nuclei. Neuropsychopharmacology.

[48] Rummel C. (2016). Inflammatory transcription factors as activation markers and functional readouts in immune-to-brain communication. Brain Behav. Immun..

[49] Damm J., Luheshi G.N., Gerstberger R., Roth J., Rummel C. (2011). Spatiotemporal nuclear factor interleukin-6 expression in the rat brain during lipopolysaccharide-induced fever is linked to sustained hypothalamic inflammatory target gene induction. J. Comp. Neurol..

[50] Damm J., Roth J., Gerstberger R., Rummel C. (2017). The use of siRNA as a pharmacological tool to assess a role for the transcription factor NF-IL6 in the brain under in vitro and in vivo conditions during LPS-induced inflammatory stimulation. J. Basic Clin. Physiol. Pharmacol..

[51] Schneiders J., Fuchs F., Damm J., Herden C., Gerstberger R., Soares D.M., Roth J., Rummel C. (2015). The transcription factor nuclear factor interleukin 6 mediates pro- and anti-inflammatory responses during LPS-induced systemic inflammation in mice. Brain Behav. Immun..

[52] Angus D.C., Yang L., Kong L., Kellum J.A., Delude R.L., Tracey K.J., Weissfeld L., Gen I.M.S.I. (2007). Circulating high-mobility group box 1 (HMGB1) concentrations are elevated in both uncomplicated pneumonia and pneumonia with severe sepsis. Crit. Care Med..

[53] Silva E., Arcaroli J., He Q., Svetkauskaite D., Coldren C., Nick J.A., Poch K., Park J.S., Banerjee A., Abraham E. (2007). HMGB1 and LPS induce distinct patterns of gene expression and activation in neutrophils from patients with sepsis-induced acute lung injury. Intensive Care Med..

[54] Sass G., Heinlein S., Agli A., Bang R., Schumann J., Tiegs G. (2002). Cytokine expression in three mouse models of experimental hepatitis. Cytokine.

[55] Guazzi S., Strangio A., Franzi A.T., Bianchi M.E. (2003). HMGB1, an architectural chromatin protein and extracellular signalling factor, has a spatially and temporally restricted expression pattern in mouse brain. Gene Expr. Patterns.

[56] Li Y., Li X., Qu Y., Huang J., Zhu T., Zhao F., Li S., Mu D. (2017). Role of HMGB1 translocation to neuronal nucleus in rat model with septic brain injury. Neurosci. Lett..

[57] Kim J.B., Lim C.M., Yu Y.M., Lee J.K. (2008). Induction and subcellular localization of high-mobility group box-1 (HMGB1) in the postischemic rat brain. J. Neurosci. Res..

[58] Alsaegh H., Eweis H., Kamal F., Alrafiah A. (2021). Celecoxib Decrease Seizures Susceptibility in a Rat Model of Inflammation by Inhibiting HMGB1 Translocation. Pharmaceuticals.

[59] Sun Q., Wu W., Hu Y.C., Li H., Zhang D., Li S., Li W., Li W.D., Ma B., Zhu J.H. (2014). Early release of high-mobility group box 1 (HMGB1) from neurons in experimental subarachnoid hemorrhage in vivo and in vitro. J. Neuroinflamm..

[60] Gao T.L., Yuan X.T., Yang D., Dai H.L., Wang W.J., Peng X., Shao H.J., Jin Z.F., Fu Z.J. (2012). Expression of HMGB1 and RAGE in rat and human brains after traumatic brain injury. J. Trauma. Acute Care Surg..

[61] Pflieger F.J., Hernandez J., Schweighofer H., Herden C., Rosengarten B., Rummel C. (2018). The role of neutrophil granulocytes in immune-to-brain communication. Temperature.

[62] Mitroulis I., Kambas K., Chrysanthopoulou A., Skendros P., Apostolidou E., Kourtzelis I., Drosos G.I., Boumpas D.T., Ritis K. (2011). Neutrophil extracellular trap formation is associated with IL-1beta and autophagy-related signaling in gout. PLoS ONE.

[63] Garcia-Romo G.S., Caielli S., Vega B., Connolly J., Allantaz F., Xu Z., Punaro M., Baisch J., Guiducci C., Coffman R.L. (2011). Netting neutrophils are major inducers of type I IFN production in pediatric systemic lupus erythematosus. Sci. Transl. Med..

[64] Kim S.W., Lee J.K. (2020). Role of HMGB1 in the Interplay between NETosis and Thrombosis in Ischemic Stroke: A Review. Cells.

[65] Manda-Handzlik A., Demkow U. (2019). The Brain Entangled: The Contribution of Neutrophil Extracellular Traps to the Diseases of the Central Nervous System. Cells.

[66] Kim S.W., Lee H., Lee H.K., Kim I.D., Lee J.K. (2019). Neutrophil extracellular trap induced by HMGB1 exacerbates damages in the ischemic brain. Acta Neuropathol. Commun..

[67] Hakkim A., Furnrohr B.G., Amann K., Laube B., Abed U.A., Brinkmann V., Herrmann M., Voll R.E., Zychlinsky A. (2010). Impairment of neutrophil extracellular trap degradation is associated with lupus nephritis. Proc. Natl. Acad. Sci. USA.

[68] Meurer M., Ohlmann S., Bonilla M.C., Valentin-Weigand P., Beineke A., Hennig-Pauka I., Schwerk C., Schroten H., Baums C.G., Kockritz-Blickwede M.V. (2020). Role of Bacterial and Host DNases on Host-Pathogen Interaction during Streptococcus suis Meningitis. Int. J. Mol. Sci..

[69] Price C.J., Hoyda T.D., Ferguson A.V. (2008). The area postrema: A brain monitor and integrator of systemic autonomic state. Neuroscientist.

[70] Ohnishi M., Monda A., Takemoto R., Fujimoto Y., Sugitani M., Iwamura T., Hiroyasu T., Inoue A. (2014). High-mobility group box 1 up-regulates aquaporin 4 expression via microglia-astrocyte interaction. Neurochem. Int..

[71] Rosciszewski G., Cadena V., Auzmendi J., Cieri M.B., Lukin J., Rossi A.R., Murta V., Villarreal A., Reines A., Gomes F.C.A. (2019). Detrimental Effects of HMGB-1 Require Microglial-Astroglial Interaction: Implications for the Status Epilepticus -Induced Neuroinflammation. Front. Cell. Neurosci..

[72] Sakaguchi S., Furusawa S. (2006). Oxidative stress and septic shock: Metabolic aspects of oxygen-derived free radicals generated in the liver during endotoxemia. FEMS Immunol. Med. Microbiol..

[73] Lian Y.J., Gong H., Wu T.Y., Su W.J., Zhang Y., Yang Y.Y., Peng W., Zhang T., Zhou J.R., Jiang C.L. (2017). Ds-HMGB1 and fr-HMGB induce depressive behavior through neuroinflammation in contrast to nonoxid-HMGB1. Brain Behav. Immun..

[74] Luan Z.G., Zhang H., Yang P.T., Ma X.C., Zhang C., Guo R.X. (2010). HMGB1 activates nuclear factor-kappaB signaling by RAGE and increases the production of TNF-alpha in human umbilical vein endothelial cells. Immunobiology.

[75] Feldman P., Due M.R., Ripsch M.S., Khanna R., White F.A. (2012). The persistent release of HMGB1 contributes to tactile hyperalgesia in a rodent model of neuropathic pain. J. Neuroinflamm..

[76] Allette Y.M., Due M.R., Wilson S.M., Feldman P., Ripsch M.S., Khanna R., White F.A. (2014). Identification of a functional interaction of HMGB1 with Receptor for Advanced Glycation End-products in a model of neuropathic pain. Brain Behav. Immun..

[77] Balosso S., Liu J., Bianchi M.E., Vezzani A. (2014). Disulfide-containing high mobility group box-1 promotes N-methyl-D-aspartate receptor function and excitotoxicity by activating Toll-like receptor 4-dependent signaling in hippocampal neurons. Antioxid. Redox Signal..

[78] Sha Y., Zmijewski J., Xu Z., Abraham E. (2008). HMGB1 develops enhanced proinflammatory activity by binding to cytokines. J. Immunol..

[79] Tian J., Avalos A.M., Mao S.Y., Chen B., Senthil K., Wu H., Parroche P., Drabic S., Golenbock D., Sirois C. (2007). Toll-like receptor 9-dependent activation by DNA-containing immune complexes is mediated by HMGB1 and RAGE. Nat. Immunol..

[80] Wang H., Ward M.F., Sama A.E. (2009). Novel HMGB1-inhibiting therapeutic agents for experimental sepsis. Shock.

[81] Youn J.H., Oh Y.J., Kim E.S., Choi J.E., Shin J.S. (2008). High mobility group box 1 protein binding to lipopolysaccharide facilitates transfer of lipopolysaccharide to CD14 and enhances lipopolysaccharide-mediated TNF-alpha production in human monocytes. J. Immunol..

[82] Youn J.H., Kwak M.S., Wu J., Kim E.S., Ji Y., Min H.J., Yoo J.H., Choi J.E., Cho H.S., Shin J.S. (2011). Identification of lipopolysaccharide-binding peptide regions within HMGB1 and their effects on subclinical endotoxemia in a mouse model. Eur. J. Immunol..

[83] Qin Y.H., Dai S.M., Tang G.S., Zhang J., Ren D., Wang Z.W., Shen Q. (2009). HMGB1 enhances the proinflammatory activity of lipopolysaccharide by promoting the phosphorylation of MAPK p38 through receptor for advanced glycation end products. J. Immunol..

[84] Ivanov S., Dragoi A.M., Wang X., Dallacosta C., Louten J., Musco G., Sitia G., Yap G.S., Wan Y., Biron C.A. (2007). A novel role for HMGB1 in TLR9-mediated inflammatory responses to CpG-DNA. Blood.

[85] Li S., Luo C., Yin C., Peng C., Han R., Zhou J., He Q., Zhou J. (2013). Endogenous HMGB1 is required in endotoxin tolerance. J. Surg. Res..

[86] Nurnberger F., Leisengang S., Ott D., Murgott J., Gerstberger R., Rummel C., Roth J. (2021). Manifestation of lipopolysaccharide-induced tolerance in neuro-glial primary cultures of the rat afferent somatosensory system. Inflamm. Res..

[87] Aneja R.K., Tsung A., Sjodin H., Gefter J.V., Delude R.L., Billiar T.R., Fink M.P. (2008). Preconditioning with high mobility group box 1 (HMGB1) induces lipopolysaccharide (LPS) tolerance. J. Leukoc. Biol..

[88] Huang Y.Y., Su W., Zhu Z.W., Tang L., Hu X.Q., Zhou S.H., Fang Z.F., Li J. (2016). Elevated serum HMGB1 in pulmonary arterial hypertension secondary to congenital heart disease. Vascul. Pharmacol..

[89] Wang C., Miao Y., Wu X., Huang Y., Sun M., Zhu Y., Zheng F., Sun W., Dong L. (2016). Serum HMGB1 Serves as a Novel Laboratory Indicator Reflecting Disease Activity and Treatment Response in Ankylosing Spondylitis Patients. J. Immunol. Res..

[90] Zhong H., Li X., Zhou S., Jiang P., Liu X., Ouyang M., Nie Y., Chen X., Zhang L., Liu Y. (2020). Interplay between RAGE and TLR4 Regulates HMGB1-Induced Inflammation by Promoting Cell Surface Expression of RAGE and TLR4. J. Immunol..

[91] Rummel C., Bredehoft J., Damm J., Schweighofer H., Peek V., Harden L.M. (2016). Obesity Impacts Fever and Sickness Behavior During Acute Systemic Inflammation. Physiology.

[92] Yu M., Huang H., Dong S., Sha H., Wei W., Liu C. (2019). High mobility group box-1 mediates hippocampal inflammation and contributes to cognitive deficits in high-fat high-fructose diet-induced obese rats. Brain Behav. Immun..

[93] Rummel C., Hubschle T., Gerstberger R., Roth J. (2004). Nuclear translocation of the transcription factor STAT3 in the guinea pig brain during systemic or localized inflammation. J. Physiol..

[94] Rummel C., Inoue W., Sachot C., Poole S., Hubschle T., Luheshi G.N. (2008). Selective contribution of interleukin-6 and leptin to brain inflammatory signals induced by systemic LPS injection in mice. J. Comp. Neurol..

[95] Ott D., Murgott J., Rafalzik S., Wuchert F., Schmalenbeck B., Roth J., Gerstberger R. (2010). Neurons and glial cells of the rat organum vasculosum laminae terminalis directly respond to lipopolysaccharide and pyrogenic cytokines. Brain Res..

[96] Manivannan S., Marei O., Elalfy O., Zaben M. (2021). Neurogenesis after traumatic brain injury-The complex role of HMGB1 and neuroinflammation. Neuropharmacology.

[97] Zhang J., Hua X.F., Gu J., Chen F., Gu J., Gong C.X., Liu F., Dai C.L. (2020). High Mobility Group Box 1 Ameliorates Cognitive Impairment in the 3xTg-AD Mouse Model. J. Alzheimer’s Dis. JAD.

[98] Aneja R.K., Alcamo A.M., Cummings J., Vagni V., Feldman K., Wang Q., Dixon C.E., Billiar T.R., Kochanek P.M. (2019). Lack of Benefit on Brain Edema, Blood-Brain Barrier Permeability, or Cognitive Outcome in Global Inducible High Mobility Group Box 1 Knockout Mice Despite Tissue Sparing after Experimental Traumatic Brain Injury. J. Neurotrauma..

[99] Bazinet R.P., Laye S. (2014). Polyunsaturated fatty acids and their metabolites in brain function and disease. Nat. Rev. Neurosci..

[100] Masson G.S., Nair A.R., Silva Soares P.P., Michelini L.C., Francis J. (2015). Aerobic training normalizes autonomic dysfunction, HMGB1 content, microglia activation and inflammation in hypothalamic paraventricular nucleus of SHR. Am. J. Physiol. Heart Circ. Physiol..

[101] Evran S., Calis F., Akkaya E., Baran O., Cevik S., Katar S., Gurevin E.G., Hanimoglu H., Hatiboglu M.A., Armutak E.I. (2020). The effect of high mobility group box-1 protein on cerebral edema, blood-brain barrier, oxidative stress and apoptosis in an experimental traumatic brain injury model. Brain Res. Bull..

[102] An J.Y., Pang H.G., Huang T.Q., Song J.N., Li D.D., Zhao Y.L., Ma X.D. (2018). AG490 ameliorates early brain injury via inhibition of JAK2/STAT3-mediated regulation of HMGB1 in subarachnoid hemorrhage. Exp. Ther. Med..

[103] Paxinos G., Watson C. (1998). The Rat Brain in Stereotaxic Coordinates.

[104] Ott D., Wuchert F., Murgott J., Rummel C., Gerstberger R., Roth J. (2012). The viral mimetic polyinosinic:polycytidylic acid (poly I:C) induces cellular responses in primary cultures from rat brain sites with an incomplete blood-brain barrier. Neurosci. Lett..

[105] Salm P., Taylor P.J., Kostner K. (2011). Simultaneous quantification of total eicosapentaenoic acid, docosahexaenoic acid and arachidonic acid in plasma by high-performance liquid chromatography-tandem mass spectrometry. Biomed. Chromatogr..

[106] Cao H., Xiao L., Park G., Wang X., Azim A.C., Christman J.W., van Breemen R.B. (2008). An improved LC-MS/MS method for the quantification of prostaglandins E(2) and D(2) production in biological fluids. Anal. Biochem..

