# Supplementary material for: LPS Primes Brain Responsiveness to High Mobility Group Box-1 Protein
Source: Pharmaceuticals (Basel). 2021 Jun 11;14(6):558. doi: 10.3390/ph14060558 (PMC8230749; doi:10.3390/ph14060558)
Supplement: Supplementary file 1 [file pharmaceuticals-14-00558-s001.zip › pharmaceuticals-1225193-supplementary.pdf]

pharmaceuticals

MDPI

Suppl. Figures

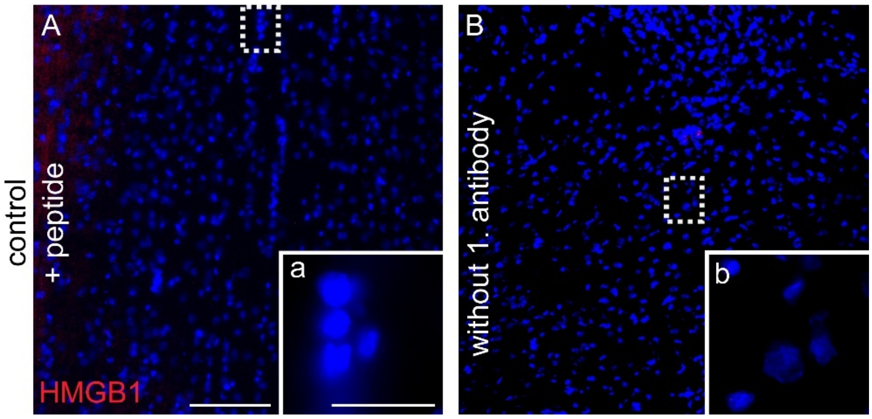

Suppl. Figure S1. Technical controls for immunohistochemical localization of HMGB1 within the median preoptic nucleus (A, MnPO) and area postrema (B, AP). 24 hours after injection of PBS or LPS, brains were isolated and frozen for later immunohistochemistry to localize HMGB1 expression (red). Specific staining disappeared in technical controls (A-B). Inserted boxes represent magnified images of marked areas (white dashed rectangles). DAPI was used to stain nuclei (blue). Scale bars represent 100 µm (A) or 25 µm (a) and are applicable for all images.

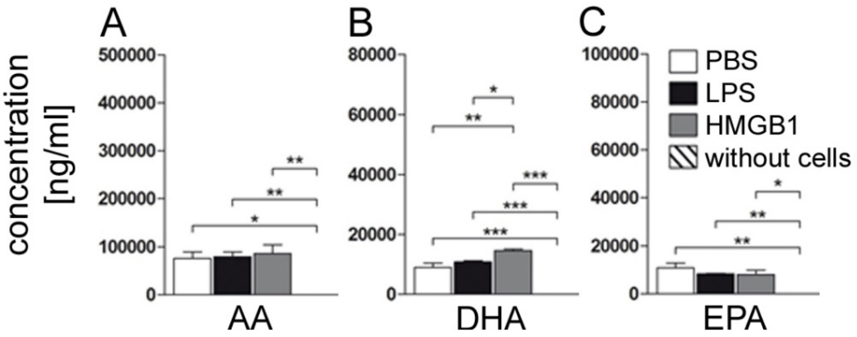

Suppl. Figure S2. Stimulation with HMGB1 increases the release of docosahexaenoic acid (DHA) from cultures of the area postrema (AP). The concentration of the fatty acids arachidonic acid (AA), DHA and eicosapentaenoic acid (EPA) was analyzed in supernatants of AP neuroglial cultures after stimulation with LPS (10 µg/mL), HMGB1 (10µg/mL) or PBS for 48 h using LC MS (A-C). All fatty acids were detectable in significantly higher concentrations than in the "blank" medium samples without cells. HMGB1 treatment significantly increased the DHA concentration 48 after stimulation (B). Three pooled samples of independent experiments were analyzed. Bars represent means ± SEM (one-way ANOVA followed by Tukey multiple comparison tests). * $p < 0.05$; ** $p < 0.01$; *** $p < 0.001$.

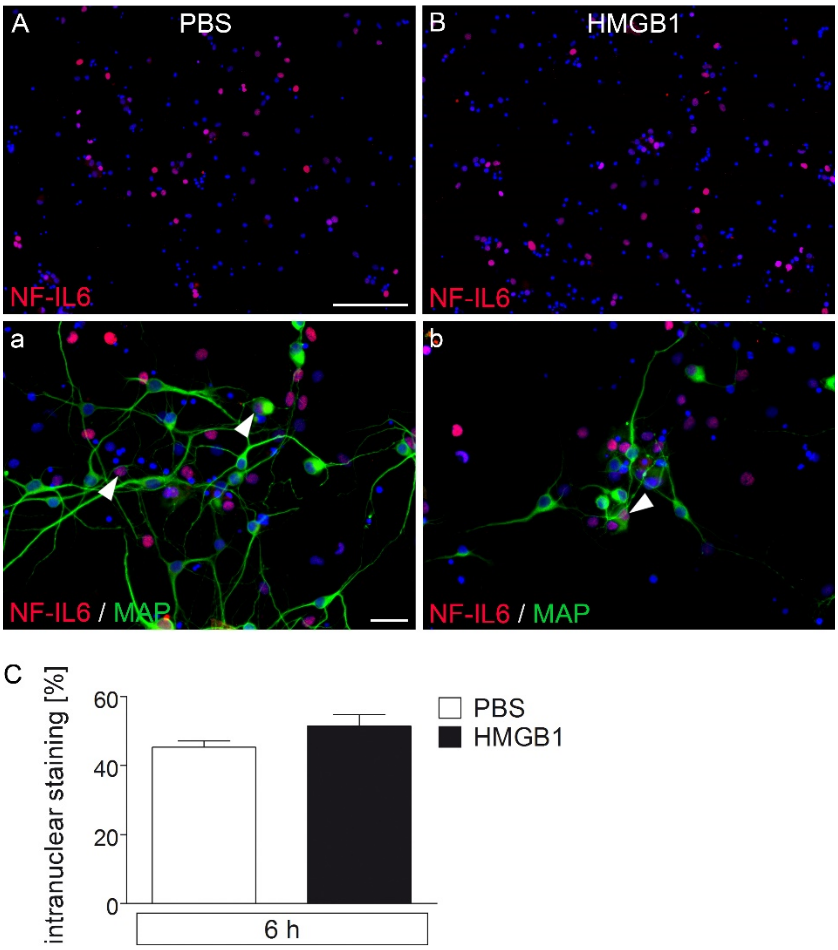

Suppl. Figure S3. HMGB1-treatment did not significantly alter NF-IL6-activation in primary cultures of the area postrema (AP). HMGB1-treatment (5 µg/mL) for six hours did not change the number of nuclear (blue, DAPI) NF-IL6-immunoreactivity (red) in primary neuroglial AP-cultures compared to PBS controls (A-B) primarily seen in non-neuronal MAP2ab-positive (green) cells (a-b). The scale bar represents 100 µm in A and applies for A-B; 25 µm for a-b. The percentage of NF-IL6-positive cells out of the total number of counted nuclei are shown as mean of the mean. A total number of 1130 PBS-incubated cells (eleven wells from five independent experiments) and 920 HMGB1-treated cells (ten wells from five independent experiments) were analyzed. Bars represent means ± SEM (t-test).

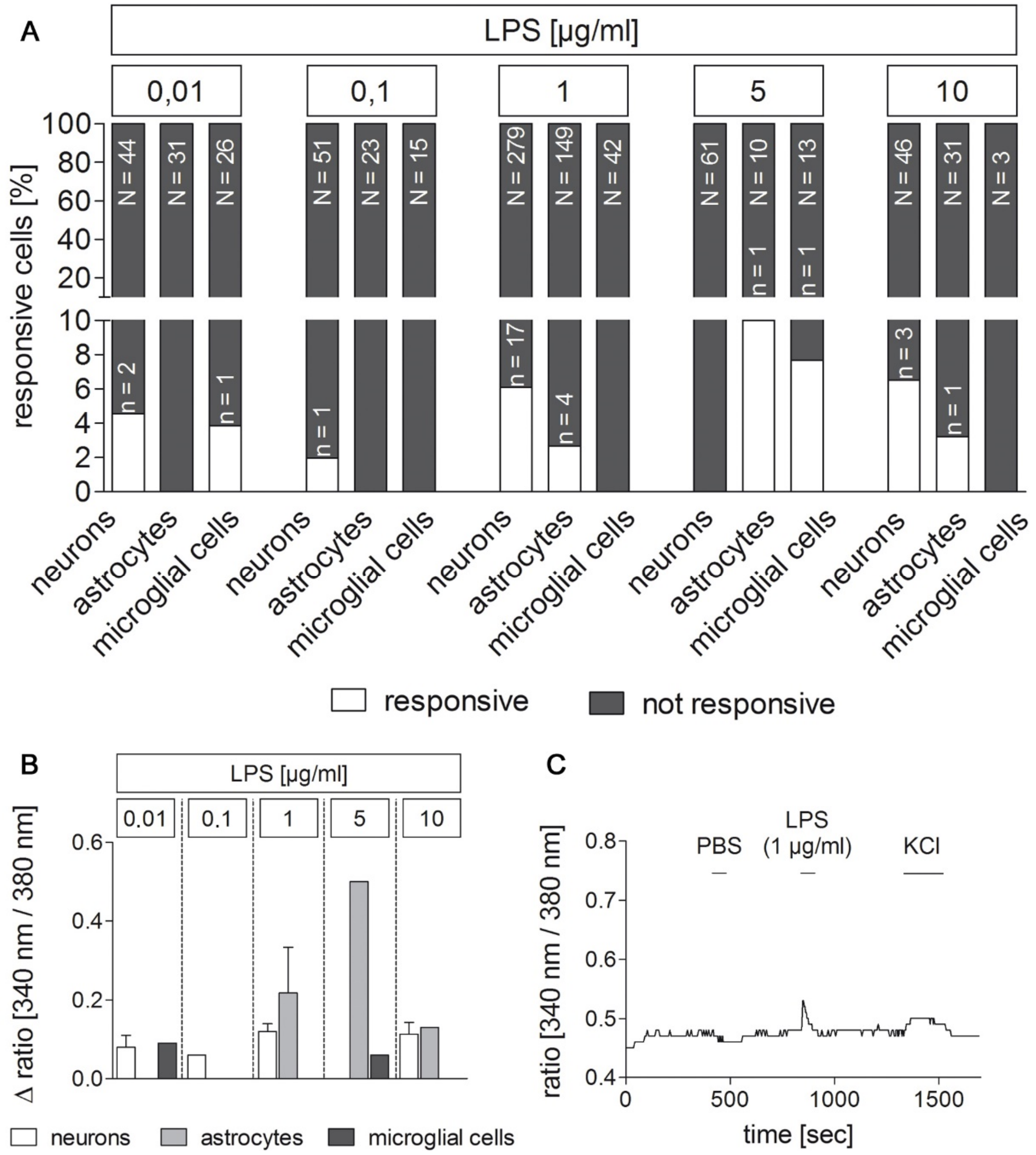

Suppl. Figure S4. Responses of cultured area postrema (AP) cells upon stimulation with LPS in Ca^{2+}-imaging experiments. Primary cell cultures of the rat AP were used for Ca^{2+}-imaging experiments 4-5 days after preparation. LPS was applied in serial dilutions (0.01, 0.1, 1, 5, 10 µg/mL) to identify stimulus-induced changes in the ratio [340/380nm] indicative for changes in intracellular calcium concentrations $[Ca^{2+}]_i$. Cell types were characterized by immunocytochemistry after each experiment. Two to 10 percent of neurons, a few astrocytes and only one microglial cell responded to LPS stimulation with an increase in $[Ca^{2+}]_i$, (A, n = responsive cells, white bars; N = amount of investigated cells, grey). Mean Δratios [340/380 nm] ± SEM of responsive cells are shown in B. A representative example of an HMGB1 responsive cell is presented in C. PBS was applied as control, while KCl (50 mM) was used to confirm neuronal viability. The numbers of investigated cultures and independent experiments are provided in suppl. Table S1.

Suppl. Table S1. Number of wells and independent experiments for the Ca^{2+} imaging experiments with LPS.

LPS	Number of wells	Number of independent cultures
0,01 µg/mL	4	2
0,1 µg/mL	4	2
1 µg/mL	19	10
5 µg/mL	4	3
10 µg/mL	5	2

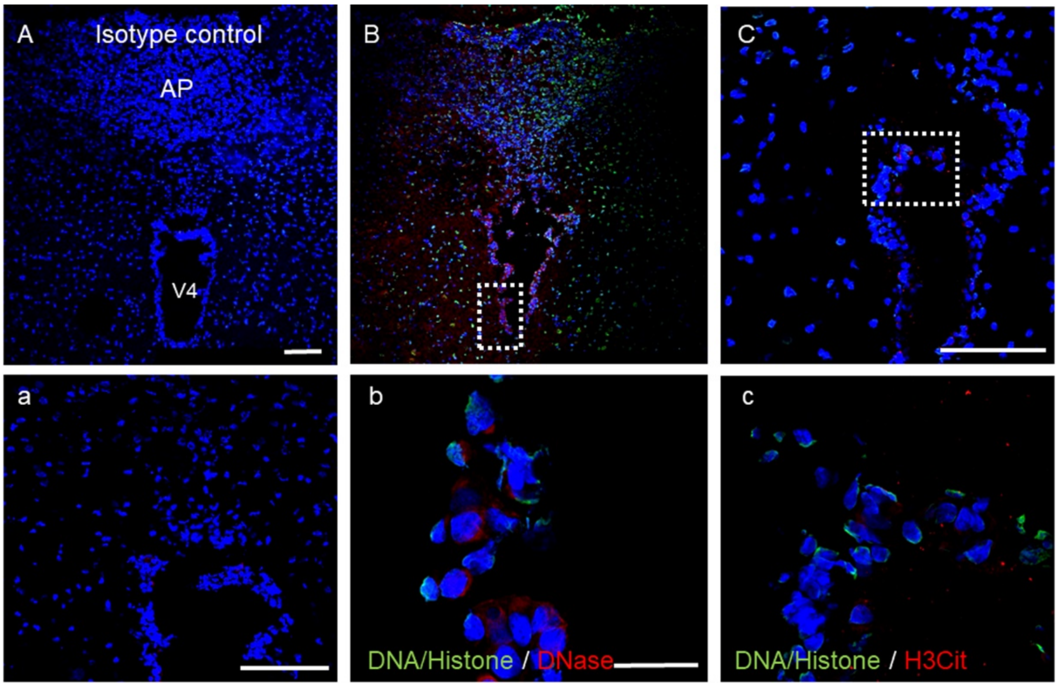

Suppl. Figure S5. Immunofluorescence staining of extacellular trap- markers and DNase in the area postrema (AP) 8 hours after LPS-stimulation. 8 hours after injection of LPS [1 mg/kg], brains were isolated and frozen for later immunofluorescence staining to localize DNA/Histone (green) combined with H3Cit or DNAse1 (red). Isotype controls confirmed specificity (A, a). Rats treated with LPS showed typical DNA/Histone (green) and H3Cit (red, C, c) staining indicative of extra-cellular trap formation (ETs). As a reaction of ET formation, the host cells release DNase (red, B, b). Lower case labelled microphotographs represent magnified images of marked areas (white dashed rectangles). Representative microphotographs are shown out of four LPS-treated mice. Scale bars represent 100 µm (A-C, a) or 25 µm (b-c).
